# Endocrine system disturbances in children with inherited metabolic diseases: a narrative review

**DOI:** 10.3389/fendo.2025.1717675

**Published:** 2025-12-17

**Authors:** Veronica Maria Tagi, Laura Fiori, Chiara Montanari, Davide Tonduti, Matilde Ferrario, Mirko Gambino, Ilenia Pia Greco, Alessandra Cecchini, Valeria Calcaterra, Gianvincenzo Zuccotti, Elvira Verduci

**Affiliations:** 1 Department of Pediatrics, Vittore Buzzi Children’s Hospital, Milan, Italy; 2 Department of Biomedical and Clinical Science, University of Milan, Milan, Italy; 3 COALA (Center for Diagnosis and Treatment of Leukodystrophies), Unit of Pediatric Neurology, Vittore Buzzi Children’s Hospital, Milan, Italy; 4 Pediatric and Adolescent Unit, Department of Internal Medicine, University of Pavia, Pavia, Italy; 5 Department of Health Sciences, University of Milan, Milan, Italy; 6 Metabolic Diseases Unit, Department of Pediatrics, Vittore Buzzi Children’s Hospital, Milan, Italy

**Keywords:** adrenal glands, children, endocrine system, gonads, inherited metabolic diseases, pancreas, pituitary gland, thyroid

## Abstract

Inborn metabolic diseases (IMDs) represent a diverse and complex group of rare disorders, typically resulting from variants in genes that encode specific enzymes or cofactors, leading to reduced or absent enzymatic activity. These conditions commonly disrupt one or more metabolic pathways, often impacting multiple organ systems from early childhood. Clinicians should consider the possibility of an IMD when an endocrine abnormality is accompanied by other unexplained clinical signs or in presence of combined endocrinopathies. While some IMDs associated with endocrine dysfunction in children and adolescents are well-documented and supported by established treatment guidelines, others lack clear recommendations or are characterized by inconsistent data. This narrative review aims to summarize the main IMDs that present with endocrine abnormalities in pediatric patients, organized according to affected organ systems and underlying pathophysiological mechanisms. Furthermore, we reviewed the latest recommendations, when available, for monitoring endocrine function in children with these disorders and eventually for providing a tailored treatment, where applicable.

## 1 Introduction

**Figure 1:**
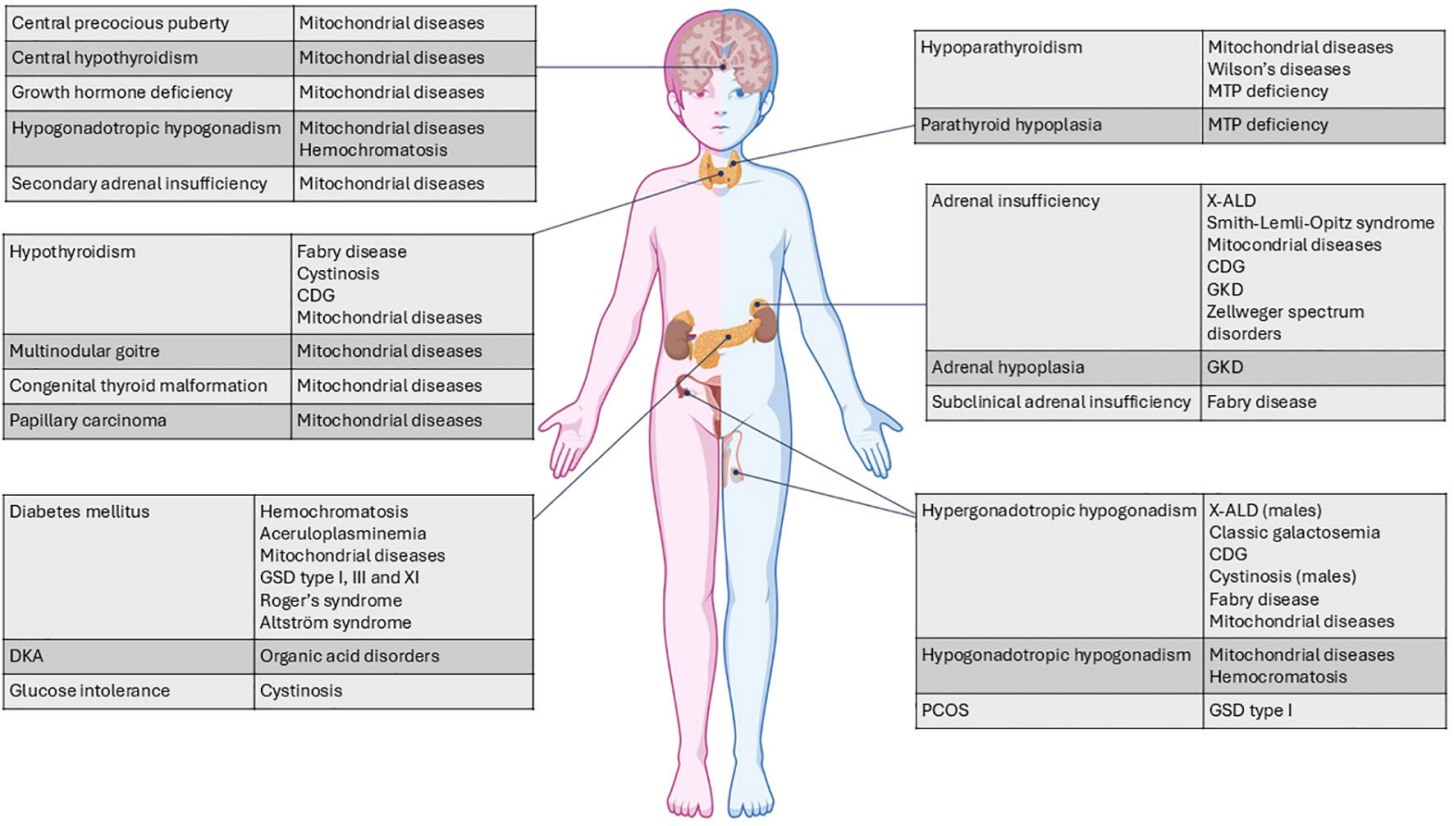
Main endocrine systems and organs involved in IMDs and relative clinical manifestations. CDG, congenital disorders of glycosylation; DKA, diabetic ketoacidosis; MTP, mitochondrial trifunctional protein; X-ALD, X-linked adrenoleukodystrophy; GKD, glycerol kinase deficiency; PCOS, polycystic ovary syndrome; POI, premature ovarian insufficiency; GSD, glycogen storage disease. This figure was created using BioRender.com.

Inborn metabolic diseases (IMDs) are a large and heterogeneous group of rare disorders, usually caused by variants in genes encoding specific enzymes or cofactors, leading to an impairment of their activity (1). Since each of these diseases is due to the dysfunction of one or more metabolic pathways, they often affect multiple organs since childhood, although a single endocrinopathy may also occur (2). **Figure 1** illustrates the main endocrine systems and organs involved in IMDs.

IMDs should be suspected particularly in children presenting with combined endocrinopathies. Mitochondrial diseases are a typical example of IMDs with frequently reported combined endocrinopathies (3, 4).

The suspicion of an inherited metabolic disease should arise even in case of an endocrinopathy associated with other clinical features which are not explained by the endocrinopathy itself. For instance, diabetes mellitus has been described in several IMDs, such as hemochromatosis, aceruloplasminemia, mitochondrial diseases, and GSD type I and III (3, 5–8). Therefore, even in case of a common endocrine disorder in pediatric patients, a complete clinical examination and critical evaluation of blood and, when appropriate, radiological studies should be performed. In the presence of clinical, radiological, or biochemical findings that are not typical of the diagnosed endocrinological pathology, the appropriate investigations should be carried out in a multidisciplinary context to exclude an underlying rare disease.

While for some IMDs the endocrine system disturbances in children or adolescents are extensively described in the literature, together with well-defined treatment guidelines, for others data remain controversial or clear management recommendations are lacking.

In this narrative review we specifically address the following research question: which endocrine manifestations are associated with IMDs, and through which underlying pathophysiological mechanism? Therefore, our first aim is to provide an overview of the main IMDs described with a primary endocrine impairment from childhood or adolescence, exploring the underlying pathogenetic mechanisms by organ. Our second aim is to explore the most recent available recommendations for monitoring endocrine organ function in children with these diseases, together with the specific treatments, where applicable.

To the best of our knowledge, this is the first comprehensive overview of the most commonly reported endocrine manifestations of IMDs in children, as well as the available recommendations for their management in the pediatric population.

## 2 Methods

We conducted a narrative review to explore the main IMDs with reported primary endocrine involvement manifesting in children and adolescents, as well as their follow-up and management. We performed a comprehensive literature search using the PubMed (Medline) and Scopus databases, covering articles published in English between 2000 and 2025. Inclusion criteria were: original research articles, systematic and narrative reviews, guidelines, case reports, and case series describing endocrine involvement in pediatric IMDs; IMDs with documented primary endocrine manifestations (direct effect on endocrine organs). Although the primary focus is on the pediatric population, in a few IMDs we cite evidence derived from adult cohorts only when pediatric data were not available, and these instances are clearly indicated in the text.

*Search strategy*

As research strategy, we used the following keywords: “inherited metabolic diseases”, “inborn errors of metabolism”, “disorders of fatty acid metabolism”, “disorders of amino acid metabolism”, “disorders of carbohydrate metabolism”, “lysosomal storage disorders”, “congenital disorders of glycosylation”, “mitochondrial diseases”, “organic acidurias”, “disorders of metal metabolism”, “urea cycle disorders”. These were combined with the terms “endocrine system”, “thyroid gland”, “parathyroid glands”, “gonads”, “adrenal glands”, “pancreas”, “pituitary gland” and “etiopathogenesis” for our first aim, or with “management”, “treatment”, “follow-up” for our second aim.

Starting from a total of 312 papers, 71 were excluded after an initial screening based on titles and abstracts. The full texts of the remaining articles were then reviewed, and 147 relevant papers were selected for detailed analysis and critical discussion. References from all selected articles were also checked for additional relevant studies.

**Figure 2:**
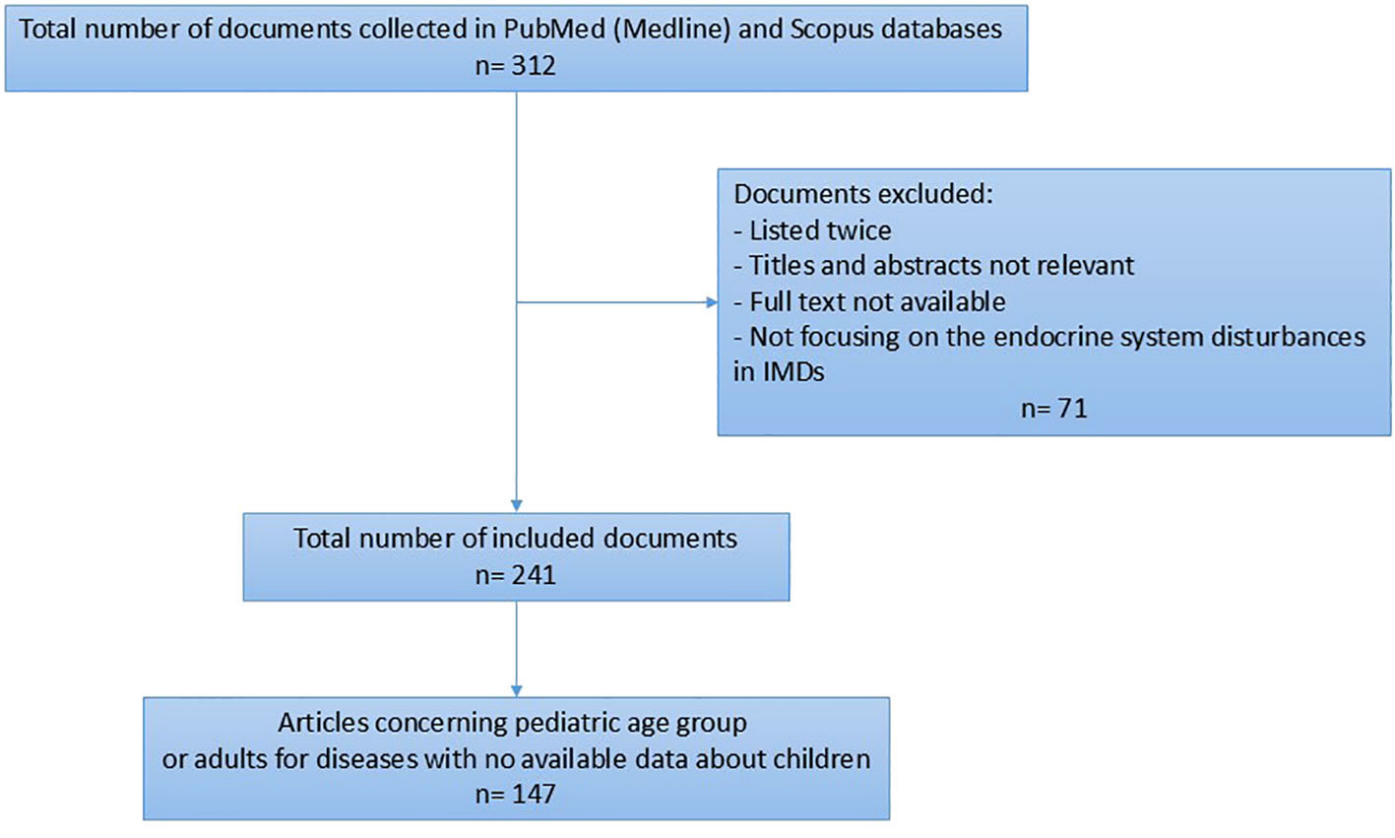
Flow chart showing the process of articles selection and exclusion. Starting from a total of 312 papers, 71 articles were excluded by a first screening. The authors then reviewed the full texts of the remaining papers and finally selected 147 relevant articles, which were analyzed to develop a critical discussion.

A flow diagram illustrating the process of paper selection and exclusion is presented in **Figure 2**.

## 3 Results

### 3.1 Thyroid gland

Thyroid gland function may be impaired in several IMDs, through different pathogenetic mechanisms (**Table 1**).

**Table 1:** Reported involvement of the thyroid gland in various IMDs, with recommendations for monitoring and treatment.

IMD	Endocrine disturbances	Suggested follow-up	Treatment options
Fabry disease	Primary hypothyroidism (9, 10)	Regular TSH monitoring since childhood (9)	Hormonal replacement (9) Enzyme Replacement Therapy
Cystinosis	Primary hypothyroidism (12–14, 16)	Regular TSH monitoring since early childhood (14, 16)	Hormonal replacement (14, 15) Cysteamine
CDG	Hypothyroidism (17, 18)	Regular TSH and FT4 assessment every six months during the first two years of life and annually thereafter (17, 18) and in critical circumstances (hypoalbuminemia, sepsis, protein-losing enteropathy, etc.) (17, 20)	Thyroid hormone replacement treatment in case of decreased FT4 levels combined with altered TSH levels (17, 18, 20).
Mitochondrial diseases	Primary hypothyroidism (mtDNA) (21–24) (3, 134) Multinodular goitre, congenital thyroid malformation and papillary carcinoma (KSS) (22)	Thyroid function monitoring since childhood (21)	No specific recommendations available

IMD, inherited metabolic disease; CDG, congenital disorders of glycosylation; mtDNA, mitochondrial DNA; KSS, Kearns-Sayre syndrome; TSH, thyroid-stimulating hormone; FT4, free thyroxine.

Fabry disease (FD) is a rare X-linked lysosomal storage disease caused by variants in the *GLA* gene leading to reduced activity of the encoded lysosomal enzyme alfa-galactosidase A. Consequently, glycosphingolipids (in particular, globotriaosylceramide [Gb3] and globotriaosylsphingosine [lyso-Gb3]) accumulate in lysosomes causing diffuse organ damage. In FD, endocrine organs such as the thyroid gland are among the targets for glycosphingolipid accumulations due to their high vascularization and low proliferation rate. However, the underlying pathophysiology of hypothyroidism observed in patients with FD remains unclear, since it is not known whether these accumulations are responsible for thyroid dysfunction (9, 10). Although no specific follow-up guidelines exist, the relatively high incidence of subclinical hypothyroidism supports regular thyroid function screening from childhood, with initiation of levothyroxine replacement when dysfunction is detected (9, 10). Results from the observational study by Faggiano et al. (2011), conducted on 14 FD patients (7 females, 7 males) aged 21–62 years, suggest that primary hypothyroidism in FD patients may be reversed after long-term enzyme replacement therapy (ERT) (11).

Cystinosis is a rare hereditary disorder resulting from variants in the *CTNS* gene. This gene encodes the carrier protein cystinosin, responsible for transporting cystine out of lysosomes. Defective cystinosin function leads to widespread accumulation of cystine crystals in all body cells and tissues, including the thyroid gland. Progressive storage of cystine in thyroid follicular cells results in fibrosis and atrophy, leading to primary hypothyroidism (12). This endocrine condition affects the majority of patients with cystinosis by the second decade of life (12). Early thyroid changes affecting thyroglobulin synthesis and iodothyroglobulin processing may cause subclinical hypothyroidism, characterized by elevated TSH and normal T3 and T4 levels. Hormone replacement therapy is crucial for managing hypothyroidism in cystinosis; therefore, careful monitoring of thyroid function is mandatory in patients with this disease. Since hypothyroidism may manifest during the first years of life (13), thyroid function monitoring is recommended from early childhood (14). Although levothyroxine dosing guidelines are not available, treatment is generally required (14, 15) The disease-specific treatment, aminothiol cysteamine, acts by depleting lysosomal cystine, thereby also reducing the need for thyroid hormone replacement therapy (12, 14, 16).

Congenital disorders of glycosylation (CDG) are a group of rare genetic disorders that include defects in synthetic pathways for N-linked oligosaccharides, O-linked oligosaccharides, shared substrates, glycophosphatidylinositol (GPI) anchors, and dolichols. They are characterized by abnormal glycosylation, resulting in multisystem involvement and a heterogeneous clinical presentation (17). Glycosylation is a crucial process in the synthesis and function of various proteins, including those involved in thyroid hormone production and regulation. It has been postulated that disruptions in glycosylation processes may potentially affect thyroid-related proteins and pathways (18). Structural and functional alterations of glycosylated proteins in CDG individuals can make assessment of thyroid function challenging. However, despite decreased thyroid hormone levels, TSH levels are often reported to be normal (19). Clinical symptoms of hypothyroidism might resemble more severe CDG symptoms, especially during acute metabolic decompensation (18). Despite the challenges, early identification and treatment of thyroid dysfunction remain fundamental (20). Therefore, assessing thyroid function, even in critical circumstances, is reasonable, keeping in mind that an euthyroid sick syndrome may coexist. TSH and FT4 levels should be measured regularly, and thyroid hormone replacement therapy should be considered in case of altered values (17, 18, 20).

Mitochondrial diseases constitute a clinically heterogeneous group of disorders that impact mitochondrial function. A significant association between mitochondrial diseases and thyroid dysfunction has been observed, particularly in specific mitochondrial disorders (3). For example, mitochondrial DNA (mtDNA) deletion disorders, including Kearns-Sayre syndrome (KSS) and polymerase γ(POLG)-related mtDNA depletion syndrome, may present with hypothyroidism (3, 21–23). In KSS, thyroid dysfunction is reported in around 3% of cases, encompassing diverse thyroid abnormalities such as compensated hypothyroidism, multinodular goitre, congenital thyroid malformation, and papillary carcinoma (22). mtDNA depletion disorders, such as POLG-related mtDNA depletion syndrome, have been linked to a broader spectrum of endocrinopathies, including hypothyroidism, adrenal insufficiency, and diabetes (3, 24). While no curative treatment is available, addressing treatable aspects of associated endocrine dysfunctions holds the potential to improve patients’ outcomes. For this reason, a comprehensive follow-up strategy, including routine screening for thyroid function and target treatment, is imperative (3), although no specific guidelines are available in this regard.

Overall, despite the heterogeneity of underlying mechanisms, these pediatric IMDs share common challenges, including limited understanding of pathophysiology, lack of standardized screening protocols, and scarcity of pediatric-specific treatment guidelines. Addressing these gaps through focused clinical studies and longitudinal research is essential to optimize early detection, monitoring, and management of endocrine dysfunction in children with IMDs.

### 3.2 Parathyroid glands

Parathyroid glands disfunction, particularly hypoparathyroidism, has been described in patients with IMDs (**Table 2**).

**Table 2:** Reported involvement of the parathyroid glands in various IMDs, with recommendations for monitoring and treatment.

IMD	Endocrine disturbances	Suggested follow-up	Treatment options
Mitochondrial diseases	Hypoparatyroidism (KSS) (3, 21, 22)	Parathyroid hormone, calcium, magnesium and phosphate ions regular monitoring since childhood (3)	Vitamin D and calcium supplementations (21)
Wilson’s disease	Hypoparatyroidism (29–32)	No recommendations available	No specific recommendations available
MTP deficiency	Hypoparatyroidism (33–36) Parathyroid hypoplasia (36)	Periodic assessment of blood calcium, phosphorus, and parathyroid hormone levels since the first months of age (33, 34)	No recommendations available

IMD, inherited metabolic disease; MTP, mitochondrial trifunctional protein; KSS, Kearns-Sayre syndrome.

Hypoparathyroidism is notably observed in mitochondrial diseases, especially in severely affected individuals who show multisystemic involvement manifesting during childhood (3, 21). For example, in KSS, approximately 6% of cases exhibit hypoparathyroidism, often accompanied by concurrent renal tubulopathy leading to imbalances in calcium, magnesium, and potassium (22). In mitochondrial diseases, hypoparathyroidism is frequently associated with additional endocrine abnormalities such as diabetes mellitus, short stature, and gonadal dysfunction (22).

In mitochondrial diseases, including KSS, hypoparathyroidism is thought to result from a combination of renal tubular electrolyte losses and direct mitochondrial dysfunction in parathyroid cells, leading to impaired energy-dependent PTH secretion (21, 25, 26). Despite magnesium supplementation, low PTH concentrations may persist (26, 27). Although no specific guidelines are available, these evidence suggest that regular assessment of blood PTH, calcium, magnesium and phosphate levels from childhood, along with supplementation with vitamin D and calcium when needed, may help prevent hypoparathyroidism and its complications in these children (3, 21).

Wilson’s disease is a genetic disorder caused by variants in the *ATP7B* gene, located on chromosome 13. These variants lead to impaired copper excretion from the liver into bile and defective incorporation of copper into ceruloplasmin, the protein responsible for copper transport in the blood (28).

The pathogenesis of hypoparathyroidism in Wilson’s disease involves the toxic effects of excess copper deposition in various tissues (28). Wilson’s disease primarily causes copper accumulation in the liver, brain, kidneys, and other organs, but some authors have reported that excess copper may also be present in the parathyroid glands, particularly in children during the second decade of life and in young adults (29–32). According to the literature, copper deposition in the parathyroid glands may result in parenchymal degeneration, as observed in post-mortem examinations (30). This degeneration of parathyroid tissue may be analogous to iron deposition observed in thalassemia, which leads to granular involution, In Wilson’s disease, excess copper can cause hemolytic anemia through inhibition of red blood cell enzymes, and the resulting vascular and parenchymal changes may further impair parathyroid function (30). This process leads to hypoparathyroidism with secondary hypocalcemia, low serum magnesium levels, elevated serum phosphorus, and absent or inappropriately low levels of PTH (31, 32). Further studies and long-term evaluations are needed to determine the reversibility of hypoparathyroidism in Wilson’s disease and the potential impact of therapeutic interventions, given the intricate relationship between copper deposition and parathyroid function (30). While calcium supplementation has been reported to be effective in children and young adults (31, 32), no data are currently available on the effects of copper chelators on parathyroid function.

Mitochondrial trifunctional protein (MTP) is a hetero-octamer composed of four α- and four β-subunits and carries out three distinct enzyme activities that catalyze the final chain-shortening reactions in the β-oxidation of long-chain fatty acids. The *HADHA* gene encodes the α-subunit, which is involved in both long-chain enoyl-CoA hydratase (LCEH) and long-chain 3-hydroxyacyl-CoA dehydrogenase (LCHAD) activities, whereas the *HADHB* gene encodes the b-subunit, involved in the Long-chain 3-ketoacyl-CoA thiolase (LCKAT) activity. Variants in *HADHA* or *HADHB* cause MTP deficiency, resulting in decreased activity and levels of all three enzymes because of failed hetero-octamer formation. However, a homozygous variant (1528G>C) in *HADHA* has been reported to cause isolated LCHAD deficiency. Hypoparathyroidism has been reported in four children with MTP deficiency (33–35) and in one child with isolated LCHAD deficiency (36), all manifesting within the first 15 months of life.

The pathogenetic link between MTP deficiency and hypoparathyroidism remains unclear and seems to involve several complex factors. MTP deficiency disrupts the oxidation of long-chain fatty acids within mitochondria, leading to the accumulation of toxic compounds that may affect various tissues, including the parathyroid glands. The resulting mitochondrial dysfunction impacts cellular processes, potentially influencing the secretion of PTH (37). However, hypoparathyroidism has not been reported in other patients with the most common mitochondrial β-oxidation defects, such as carnitine palmitoyl transferase II (CPTII) deficiency and very-long-chain acyl-CoA dehydrogenase deficiency (VLCADD) (34). Another possible explanation for hypoparathyroidism in MTP deficiency is the presence of congenital parathyroid malformations, particularly hypoplasia, which have been described in patients with LCHAD deficiency (36). Although there are no clear guidelines regarding the clinical management of hypoparathyroidism in patients with MTP defects, the available case reports suggest that these patients require periodic evaluation of blood calcium, phosphorus, and PTH levels. A multidisciplinary approach, involving both metabolic and endocrine specialists, is essential to tailor therapy and ensure optimal monitoring (33, 34).

Despite these reports, significant gaps remain in understanding the precise mechanisms linking IMDs to hypoparathyroidism, as well as the natural history and long-term outcomes of affected children. Further research is needed to clarify disease-specific pathophysiology, assess potential reversibility with treatment, and establish evidence-based monitoring and management protocols for pediatric patients.

### 3.3 Gonads

Gonadal involvement in metabolic diseases is heterogeneous. The most frequent clinical patterns are characterized by hypogonadism with reduced fertility, ambiguous genitalia, or polycystic ovarian syndrome (PCOS) (**Table 3**).

**Table 3:** Reported involvement of the gonads and reproductive system in various IMDs, with recommendations for monitoring and treatment.

IMD	Endocrine disturbances	Suggested follow-up	Treatment options
X-ALD	Hypergonadotropic hypogonadism with normal or reduced testosterone (39–41)	No recommendations available	Testosterone replacement therapy for X-ALD males with hypogonadism signs and low testosterone (39)
Classic galactosemia	Hypergonadotropic hypogonadism in females (44, 45)	Follicle-stimulating hormone and 17-beta-estradiol assessment in girls who reach the age of 12 years with insufficient secondary sex characteristics or the age of 14 years with no regular menses (47) In women with normal pubertal development monitor for POI symptoms, with possible FSH testing (47)	In case of hypergonadotropic hypogonadism puberty induction involving step-wise escalating doses of estrogen and cyclic progesterone administration (47) Hormone replacement therapy in cases of secondary amenorrhea (47)
CDG	Hypergonadotropic hypogonadism (52)	No recommendations available	No recommendations available
Cystinosis	Pubertal delay and hypergonadotropic hypogonadism in males Pubertal delay with normal gonadal function in females (53–55)	Six-monthly assessments of growth, pubertal stage, and bone age in prepubertal males Regular assessment of LH, FSH, testosterone, inhibin B, spermiogram and testicular ultrasound in pubertal males Appropriate pre-pregnancy counselling, with assessment of thyroid function and glucose tolerance in female (53, 54)	In case of hypogonadism referral to a specialist for consideration of fertility preservation therapies In pregnant females treatments to reduce the risk of complications (e.g., acetylsalicylic acid), peripartum and postpartum respiratory support (53, 54) Cysteamine
Fabry disease	Altered LH and SHBG levels in males, menstrual abnormalities in females (10, 56) Infertility debated	Clinical monitoring, annual in boys, every 2–3 years in girls (59)	No recommendations available Enzyme Replacement Therapy
GSD type I	PCOS, dysmenorrhea and menorrhagia in girls (61, 62, 64)	Regular gynecological evaluations and specific questions about the occurrence of menorrhagia or irregular menstrual bleeding (62). In case of menhorragia, coagulation assessment and referral to a gynecologist or hematologist (64)	For menorrhagia: diet, hemostatic therapies (65, 66), hormonal treatment (67, 68), surgery (64). For PCOS: diet intervention to reduce insulin resistance and hormonal treatments (60, 63).
Mitochondrial diseases	POI (Perrault syndrome, AARS2) Hypogonadotropic hypogonadism (mtDNA, MNGIE, primary coenzyme Q10 deficiency, Leigh syndrome) (3)	No recommendations available	No recommendations available

IMD, inherited metabolic disease; X-ALD, X-linked adrenoleukodystrophy; CDG, congenital disorders of glycosylation; GSD, glycogen storage disease; mtDNA, mitochondrial DNA; MNGIE, mitochondrial neurogastrointestinal encephalomyopathy; LH, luteinizing hormone; FSH, follicle-stimulating hormone; SHBG, sex hormone-binding globulin; PCOS, polycystic ovary syndrome; POI, premature ovarian insufficiency.

X-linked adrenoleukodystrophy (X-ALD) is a peroxisomal disorder caused by variants in the *ABCD1* gene, leading to impaired cellular trafficking of very-long-chain fatty acids (VLCFA) (38). Although rare, male patients with X-ALD may present with hypogonadism with normal or reduced testosterone and elevated luteinizing hormone (LH) levels, likely due to VLCFA toxicity on testicular Sertoli and Leydig cells and potential androgen receptor resistance (39–41). Fertility reduction is uncommon, with only a few cases of declining fertility reported (39, 42, 43). Testosterone replacement therapy should be considered for X-ALD males with clinical signs of hypogonadism and low testosterone levels (39).

Most females with classic galactosemia, even with early diagnosis and good compliance to a lifelong galactose-restricted diet, develop premature ovarian insufficiency (POI), manifesting as delayed puberty, amenorrhea or oligomenorrhea, and infertility (44, 45). These complications are not observed in Duarte variant galactosemia (46). The likely underlying pathogenesis involves altered ovarian granulosa cell function and reduced anti-mullerian hormone (AMH) levels (45), possibly beginning in the prenatal period. International guidelines recommend screening for hypergonadotropic hypogonadism in girls lacking secondary sexual characteristics by the age of 12, or experiencing primary amenorrhea by the age of 14, measuring follicle-stimulating hormone (FSH), LH and 17-beta-estradiol levels (47). If hypergonadotropic hypogonadism is diagnosed, prompt referral to a pediatric endocrinologist is recommended for puberty induction using stepwise escalating doses of estrogen and cyclic progesterone administration (47). Girls with normal pubertal development should be monitored for POI symptoms, with FSH, LH, and estradiol testing as needed. Hormone replacement therapy should be initiated in cases of amenorrhea due to POI (47). Fertility preservation techniques are not routinely indicated and require individual consideration (48). In contrast, most male patients generally do not exhibit fertility issues, and routine endocrinological follow-up is generally unnecessary (47). Possible explanations for the more severe impact on females reproduction include: higher *GALT* mRNA expression in ovary and liver versus lower in testis, suggesting organ-specific vulnerability (49, 50); the ability of male testes to replenish spermatogonia lost to apoptosis, unlike females; and limited effect of LH hypoglycosylation on male reproductive function and lower reliance on FSH activity (49, 51).

Phosphomannomutase 2 deficiency (PMM2-CDG) may also lead to hypogonadism, as glycosylation is crucial for both spermatogenesis and oogenesis (52). However, literature on this topic is limited, and no standardized follow-up or treatment protocol exist.

Cystinosis can impair gonadal function as well. Males often show low testosterone, elevated FSH and LH, pubertal delay, and azoospermia. Conversely, females typically experience pubertal delay but maintain normal gonadal function, though they have increased risk of pregnancy complications, including pre-eclampsia, preterm delivery, and gestational diabetes (53–55). Prepubertal males should undergo six-monthly assessments of growth, pubertal stage, and bone age. Pubertal males should also have additional evaluations including LH, FSH, testosterone, inhibin B, AMH, and potentially a spermiogram and testicular ultrasound. Early detection of hypogonadism and pubertal delay requires referral for fertility preservation (53, 54).

Gonadal involvement in Fabry disease remains controversial. Some authors report azoospermia and infertility in males (9), while others describe alterations in sex hormone-binding globulin (SHBG) and LH in patients with chronic kidney disease (56). In females, menstrual abnormalities and miscarriages have been reported despite a high reproductive success rate (56). Conversely, other authors find no significant fertility abnormalities (10). Proposed mechanisms include glandular deposition of globotriaosylceramide (Gb3) or vascular alterations affecting testicular fluid balance and function (57, 58). Annual clinical monitoring in boys and every 2–3 years in girls is considered appropriate (59).

Glycogen storage disease (GSD) type 1 is characterized by impaired glycogenolysis and gluconeogenesis, with glycogen accumulation in tissues. Clinical manifestations include hepatomegaly, severe hypoglycemia, lactic acidosis, hyperuricemia, and hypertriglyceridemia (60). Female patients may develop PCOS, dysmenorrhea, and menorrhagia, although reproductive rates are typically preserved (61–63). The underlying pathogenesis is unclear (63). Early diagnosis and proper treatment may prevent delayed puberty (61). Recommended management includes regular gynecological evaluations, documentation of menstrual irregularities (62) and assessment of coagulation if menorrhagia is present, with referral to a gynecologist or hematologist as needed (64). Non-hormonal treatments (dietary intervention to reduce insulin resistance or hemostatic therapies like 1-desamino8-D-arginine vasopressin and antifibrinolytics) may be indicated (65, 66). Regarding hormonal treatment, due to the higher adenoma risk in glycogen storage disease type I, estrogens should be avoided. Progestin-only contraceptives are safer, but long-term medroxyprogesterone use may lower bone density, requiring close monitoring (67, 68). In girls with GSD type 1 experiencing severe menorrhagia not controlled by medical therapy, surgical interventions may be considered (64).

Hypergonadotropic hypogonadism has also been reported in patients with mitochondrial diseases, including mtDNA depletion syndromes (*C10orf2*, *POLG* variants), mitochondrial neurogastrointestinal encephalomyopathy (MNGIE), primary coenzyme Q10 deficiency, and Leigh syndrome caused by *LRPPRC* variants (69, 70). POI is also frequent in children with *AARS* variants (71). These endocrine abnormalities seem to be associated with impaired mtDNA maintenance or mitochondrial steroidogenesis (3). In some cases, POI may precede neurological symptoms (3), though no standardized recommendations exist regarding follow-up or treatment in this patient population.

Despite the extensive clinical observations, significant gaps remain in understanding the precise mechanisms leading to gonadal dysfunction across different IMDs, as well as the variability in severity between sexes and among individual diseases. Further research is needed to clarify pathophysiological pathways, assess long-term reproductive outcomes, and establish evidence-based screening and management protocols tailored to each disorder.

### 3.4 Adrenal glands

Adrenal gland involvement occurs in several congenital errors of metabolism, including X-linked adrenoleukodystrophy (X-ALD), Smith-Lemli-Opitz syndrome, mitochondrial diseases, Glycerol kinase deficiency (GKD), Fabry disease, and Congenital Disorders of Glycosylation (CDG) (**Table 4**).

**Table 4:** Reported involvement of the adrenal glands in various IMDs, with recommendations for monitoring and treatment.

IMD	Endocrine disturbances	Suggested follow-up	Treatment options
X-ALD	Adrenal insufficiency (39, 73–76)	Cortisol, ACTH, renin and electrolytes assessment as soon as possible, then every 3 to 6 months until 10 years of age and annually thereafter (73)	Replacement therapy with glucocorticoids and mineralcorticoids with same schemes as other forms of AI (39)
Smith-Lemli-Opitz syndrome	Adrenal insufficiency (80–82)	Glucose and electrolytes assessment in infants (86, 87)	Corticosteroid replacement during major stressors (e.g., surgery) (86, 87)
Mitochondrial diseases	Adrenal insufficiency (MELAS, Pearson syndrome, KSS and mtDNA) (88–93)	Regularly screen for cortisol and ACTH since infancy (3)	No recommendations available
CDG	Adrenal insufficiency (PMM2-CDG) (94)	Morning cortisol and ACTH levels assessment at least annually, low-dose ACTH stimulation test in patients with abnormal cortisol and ACTH (94)	Steroid replacement therapy in case of abnormal ACTH stimulation test (94)
GKD	Adrenal hypoplasia or adrenal insufficiency (97, 98)	Perform genetic analysis to identify the complex form associated with adrenal alterations (96–98)	In case of adrenal involvement start glucocorticoid and mineralocorticoid replacement therapy as early as possible (96–98)
Fabry disease	Subclinical adrenal insufficiency (9, 10, 77)	No recommendations available	No recommendations for AI Enzyme Replacement Therapy Chaperone Therapy

IMD, inherited metabolic disease; X-ALD, X-linked adrenoleukodystrophy; CDG, congenital disorders of glycosylation; GKD, glycerol kinase deficiency; MELAS, mitochondrial encephalomyopathy, lactic acidosis and stroke-like episodes; KSS, Kearns-Sayre syndrome; mtDNA, mitochondrial DNA; ACTH, adrenocorticotropic hormone,; AI, adrenal insufficiency.

Patients with X-ALD frequently develop adrenal insufficiency (AI), characterized by reduced cortisol synthesis and, less commonly, aldosterone deficiency, presenting with asthenia, hypotension, dehydration, hyponatremia, and hypoglycemia (39). Addison’s disease is reported as the first clinical manifestation of ALD in 38% of cases, making it the most common presenting symptom of ALD in childhood (72, 73). AI occurs in about 80% of patients and often manifests as early as the age of 3 (74), although cases of onset in infancy have been described. Screening for AI at diagnosis is recommended, keeping in mind the variability of basal cortisol and ACTH in early life (73, 75, 76). Subsequent follow-up should include fasting cortisol, glycaemia, ACTH, renin (in children >2 years), and electrolytes, every 3 to 6 months until age 10, and annually thereafter (73). In cases of adrenal dysfunction, glucocorticoid replacement therapy is indicated. Furthermore, mineralcorticoid deficiency affects 40-60% of patients with X-ALD, therefore, mineralocorticoid replacement therapy is required in case of mineralocorticoid deficiency (39, 77–79).

Smith-Lemli-Opitz syndrome is caused by 7-dehydrocholesterol reductase deficiency, leading to impaired cholesterol synthesis and accumulation of 7-dehydrocholesterol (7-DHC). Clinical severity ranges from multiple major malformations to mild phenotypes with minor anomalies and intellectual disability. In severe cases, adrenal dysfunction may occur from the neonatal period (80–82), though the literature is not uniform (83). The etiopathology of adrenal dysfunction likely involves 7-DHC accumulation in adrenal tissue, as demonstrated in autopsy studies (84). However, reported elevated ACTH levels could also be secondary to the altered basal feedback regulation of the HPA axis due to abnormal steroid precursors (83, 85). Some authors recommend screening for AI, with corticosteroid replacement during major stressors (e.g., surgery) using protocols similar to congenital adrenal hypoplasia (CAH) (83, 86, 87).

Adrenal involvement, although rare in childhood, has also been reported in mitochondrial diseases, including MELAS (mitochondrial encephalomyopathy, lactic acidosis and stroke-like episodes), Pearson syndrome, KSS, and other mtDNA deletion syndromes (88–93). The mechanism may relate to the high ATP requirements of adrenal glands, with impaired ATP production leading to reduced hormone synthesis (88). However, incidence of AI in these patients appears similar to that of the general population, suggesting mitochondrial defects alone may not be sufficient for adrenal pathology (89). AI age of onset is variable, ranging from infancy to adulthood, and usually occurs in the context of severe mitochondrial disease (77, 89). Some authors recommend regular cortisol and ACTH measurements (3), though standardized treatment guidelines are lacking.

CDG may affect adrenal function. A multicenter study on 43 PMM2-CDG patients reported AI in approximately 25%, mostly manifesting in childhood, even during the first months of life (94). This dysfunction may result from abnormal N-glycosylation of enzymes, receptors, and transport proteins involved in steroidogenesis (95). Annual assessment of morning cortisol and ACTH is recommended, with ACTH stimulation testing in cases of abnormal results, to enable early detection and initiations of steroid replacement therapy (94).

Glycerol kinase deficiency (GKD), an X-linked disorder causing elevated blood and urinary glycerol levels, can be associated with adrenal abnormalities, particularly in its complex form, involving contiguous gene deletions affecting the *GKD* locus along with CAH and/or Duchenne muscular dystrophy (*DMD*) genes (96). Affected patients may present with growth failure and salt-wasting syndrome with convulsions and hyperpigmentation in early infancy (97, 98). Early recognition of adrenal dysfunction is critical to initiate glucocorticoid and mineralocorticoid replacement therapy, with dose adjustments during stressful situations such as infections (96, 97).

Although data are limited and controversial, subclinical adrenal involvement may also occur in Fabry disease (9), with reports of reduced cortisol and elevated ACTH levels, occasionally confirmed by corticotropin stimulation tests (9, 10, 77). However, no formal recommendations exist regarding adrenal monitoring or treatment in these patients.

Zellweger spectrum disorders (ZSD) are genetic peroxisomal biogenesis disorders caused by *PEX* gene variants, leading to impaired peroxisome function and accumulation of very−long−chain fatty acids. In a cohort of 24 ZSD patients (median age 15.4 years), 7 (29%) showed primary adrenal insufficiency on ACTH stimulation testing, of which 4 were asymptomatic. The underlying mechanism is thought to involve toxic effects of elevated VLCFA (especially C26:0) on the adrenal cortex, and lifelong monitoring with regular Synacthen tests plus hydrocortisone (and fludrocortisone when needed) replacement is recommended for management (99).

### 3.5 Pancreas

Diabetes mellitus (DM) and hyperinsulinemic hypoglycemia are the most common clinical manifestations of pancreas involvement in children with IMDs (**Table 5**) (8).

**Table 5:** Reported involvement of the endocrine pancreas in various IMDs, with recommendations for monitoring and treatment.

IMD	Endocrine disturbances	Suggested follow-up	Treatment options
Hemochromatosis	Diabetes mellitus (8)	Monitor the onset of insulin insufficiency and liver cirrhosis (5, 8, 100)	Insulin and phlebotomies (5, 8, 100)
Aceruloplasminemia	Diabetes mellitus (8)	Annual glucose tolerance testing, starting at age from 15 years of age (8)	Phlebotomies and iron chelation have been demonstrated to be efficient in preventing diabetes (101, 102)
Organic acid disorders	DKA (MMA, PA, IVA, HCSD) (103, 104, 106–111)	Regular monitoring for pancreatitis onset (112)	No recommendations available
Mitochondrial diseases	Diabetes mellitus (MIDD, MELAS, KSS, Wolfram syndrome) (24, 115–118)	No recommendations available	Insulin insulin-dependent diabetes and insulin-dependent type 2 diabetes, sulphonylurea for non-insulin-dependent type 2 diabetes (3, 115, 116, 119)
GSD	Diabetes mellitus (in GSD type I and type III) (6, 7)	Close monitoring with OGTT (7)	Progressively increasing doses of insulin (7)
Roger’s syndrome	Diabetes mellitus (122, 123)	Fasting serum glucose concentration, OGTT urinalysis and assessment for clinical manifestations of poor glycemic control at least annually (124)	Oral thiamine may allow avoiding insulin therapy (123, 125), but at the onset of puberty oral glucose lowering agents or insulin therapy are usually required (125, 126)
Cystinosis	Glucose intolerance (101, 127, 128)	OGTT, HbA1c and fasting glucose assessment every 5 years	Long-term oral cysteamine administration reduces significantly the risk of developing diabetes mellitus (127)
Alström syndrome	Diabetes mellitus (129, 130)	Annual glucose tolerance test from scholar age (131)	Insulin treatment, although in case of poor glycemic control escalating doses may not be effective (131)
CDG	Hyperinsulinism (132)	No guidelines available	No guidelines available, good response to diazoxide reported (132)

IMD, inherited metabolic disease; GSD, glycogen storage disease; CDG, congenital disorders of glycosylation; MMA, methylmalonic acidemia; PA, propionic acidemia; IVA, isovaleric acidemia; HCSD, holocarboxylase synthetase deficiency; MIDD, maternally inherited diabetes and deafness; MELAS, mitochondrial encephalomyopathy, lactic acidosis and stroke-like episodes; KSS, Kearns-Sayre syndrome; DKA, diabetic ketoacidosis; OGTT, oral glucose tolerance test; HbA1c, glycated hemoglobin.

In hereditary haemochromatosis, diabetes occurs in approximately 10% of patients and results from progressive insulin resistance due to hepatic oxidative stress and increasing deposition of hemosiderin in pancreatic ß-cells (5, 100). Clinically, an initial glucose intolerance precedes insulin insufficiency. The best treatment strategy is represented by insulin and phlebotomies which can delay the need for insulin therapy, required when ß-cells are no longer able to maintain glucose homeostasis, while liver transplantation is indicated only in cases of end-stage liver disease due to iron overload and cirrhosis (5, 8, 101).

In aceruloplasminemia, diabetes mellitus (DM) represents one of the main clinical manifestations (68. 5%), caused by progressive iron deposition in pancreatic ß-cells. Phlebotomies and iron chelation may prevent DM development. In patients with aceruloplasminemia an annual glucose tolerance test, starting at 15 years of age, is recommended to detect this complication (8). Phlebotomies and iron chelation have been demonstrated to be effective in preventing diabetes (101, 102).

Some organic acidemias may present with hyperglycemia mimicking diabetic ketoacidosis with hyperglycemia, as reported in children with methylmalonic acidemia (MMA) (103–105), propionic acidemia (PA) (106, 107), isovaleric acidemia (IVA) (108–110) and holocarboxylase synthetase deficiency (HCSD) (111). The pathogenesis is unclear, however, the toxic metabolite accumulation in the pancreas seems to impair pancreatic structure or function, causing insulinopenia (8, 112).

Authors suggest that pancreatitis onset should be monitored in cases of organic acid disorders in order to promptly treat their complications (112). In some reported cases diabetic ketoacidosis (DKA) has been treated with intravenous insulin and by reducing intravenous glucose infusion, but with non-univocal results (112, 113); other authors described plasma glucose levels trending towards spontaneous normalization after treatment of the metabolic crisis (114). However, no specific guidelines about DKA in organic acidurias are available.

Several mitochondrial diseases, such as maternally inherited diabetes and deafness (MIDD), MELAS and Kearns-Sayre syndrome, are strongly associated with diabetes (24, 115–118). The pathogenesis is linked to ATP deficiency which leads ß-cells to death, as histologically confirmed by a reduction in their number. Furthermore, the lack of ATP is responsible for defective insulin secretion because it prevents the closure of potassium channels and, in addition, insulin sensitivity in skeletal muscles decreases, whereby peripheral insulin sensitivity is affected (3). Mitochondrial dysfunction may cause DM, at any age (3). It is common that patients with mitochondrial diseases and type 2 diabetes need insulin therapy, while in non-insulin-dependent patients sulphonylureas are the first-choice pharmacological treatment (3, 115, 116, 119). Macro- and microvascular complications, especially proteinuria and renal insufficiency, are more common in these patients, due to the pre-existent mitochondrial dysfunction in different organs (3, 101).

Glycogen storage diseases, in particular GSD type I and type III, may evolve into diabetes in adolescence or early adulthood (6, 7). This can be related to recurrent episodes of pancreatitis, due to hypertriglyceridemia, and insulin resistance due to hepatic and muscle dysfunction (101). Therefore, a close follow-up of GSD patients with an oral glucose tolerance test (OGTT) is recommended (7). Gradual insulin titration has been reported to be effective in controlling hyperglycemia without hypoglycemic events (7).

Fanconi–Bickel syndrome (FBS), or glycogen storage disease XI, is a rare genetic disorder caused by variants in the *GLUT2* gene, leading to impaired glucose and galactose transport, glycogen accumulation in liver and kidneys, and proximal renal tubular dysfunction. Recent studies have highlighted pancreatic involvement in children with FBS, where glycogen accumulation in pancreatic β-cells contributes to dysglycemia. The underlying mechanism appears linked to impaired glucose transport and glycogen storage in multiple tissues, including the pancreas (120). Therapeutic approaches remain largely supportive, focusing on careful glucose monitoring, dietary management, however the SGLT2 inhibitor dapagliflozin seems to be effective in reducing glycogen accumulation in the renal proximal tubule, correcting metabolic acidosis and phosphaturia, and improving kidney function in a mouse model and an adult patient (121).

Roger’s syndrome, or thiamine-responsive megaloblastic anemia (TRMA), is caused by a defect in the active transport of thiamine (THTR1) in various cells, in particular this defect causes pancreatic ß-cell apoptosis and insulin secretion impairment, leading to the development of insulin-dependent diabetes (122, 123). This disease should be suspected especially when diabetes is associated with megaloblastic anemia and/or neurosensory defects (122). Annual assessment for glucose intolerance, with fasting serum glucose concentration, OGTT and urinalysis, and for clinical manifestations of poor glycemic control is recommended (124). It has been demonstrated that pharmacological treatment with oral thiamine may allow avoidance of insulin therapy (123, 125). However, at the onset of puberty, oral glucose-lowering agents or insulin therapy are usually required (125, 126).

In cystinosis, approximately 25% of patients develop endocrine pancreatic dysfunction (101, 127), with a 50% risk of glucose intolerance by age of 18 (128). Pancreatic fibrosis leads to decreased insulin secretion (8, 101). Gahl et al. (127) demonstrated that long-term oral cysteamine administration significantly reduces the risk of developing DM.

In Alström syndrome, diabetes is common due to *ALMS1* gene-related defects in ß-cell function and peripheral insulin signaling (129). Indeed, *ALMS1* is essential at the basal body of primary cilia in β-cells, regulating glucose sensing and controlled insulin release. Loss of *ALMS1* may impair β-cell proliferation, causing inappropriate glucose-independent insulin secretion, and increasing susceptibility to β-cell death under high-glucose stress (129). In a series of 182 patients, hyperinsulinemia developed in early childhood (92%) and progressed to type 2 diabetes mellitus in 82% of those older than 16 years (130). Therefore, an annual glucose tolerance test is recommended, starting from school age (131). Insulin treatment may be effective, however, in case of poor glycemic response to treatment, increasing the insulin dose may not be effective (131).

Hypoglycemia is also a rare manifestation of PMM2-CDG and hyperinsulinism has been identified as its main cause in 43% of patients reported in literature (132). The pathophysiology of hyperinsulinism in CDG has not been defined yet, however, according to a recent systematic review conducted on 933 PMM2-CDG patients, all hyperinsulinemic patients who received diazoxide were reported to respond well (132).

Although numerous clinical presentations of pancreatic involvement have been described in IMDs, many aspects of the underlying molecular mechanisms leading to diabetes or hyperinsulinemic hypoglycemia remain unclear. The progression of pancreatic dysfunction, disease-specific risk factors, and optimal long-term monitoring strategies are still not fully defined, highlighting the need for targeted screening protocols and longitudinal studies to prevent complications and improve patient outcomes.

### 3.6 Hypothalamus and pituitary gland

The hypothalamus-pituitary system may be impaired in IMDs with various mechanisms (**Table 6**). Its involvement mainly concerns the pituitary-gonadal axis, but a dysfunction of GH/IGF1 system has also been reported.

**Table 6:** Reported involvement of the pituitary gland in various IMDs, with recommendations for monitoring and treatment.

IMD	Endocrine disturbances	Suggested follow-up	Treatment options
Mitochondrial diseases	Central precocious puberty (133) Central hypothyroidism (MELAS) (3, 134) Growth hormone deficiency (KSS, MELAS) (3, 134–136) Hypogonadotropic hypogonadism (3, 134, 153–155) Secondary adrenal insufficiency (3)	GnRH stimulation test in case of precocious puberty signs (133) GH stimulation test in case of short stature (3, 134, 135) Brain MRI in case of signs of hypothalamus-pituitary disfunction (3, 133, 134)	GnRH agonist for precocious puberty (133) Hormonal replacement in case of hypothyroidism (134) Growth hormone treatment in patients with GH deficiency (3, 136–138)
Hemochromatosis	Hypogonadotropic hypogonadism (139, 141–148)	No recommendations available	Early phlebotomy, testosterone replacement therapy in males and estrogen in females (139, 149–152)

IMD, inherited metabolic disease; MELAS, mitochondrial encephalomyopathy, lactic acidosis and stroke-like episodes; KSS, Kearns-Sayre syndrome; GnRH, gonadotropin-releasing hormone; GH, growth hormone; MRI, magnetic resonance imaging.

Central precocious puberty (PP) appears to occur more often in girls with mitochondrial diseases than in the general population, resulting in increased sex hormone levels (especially estradiol) and advanced bone age compared with chronological age. The etiopathogenetic of central PP in mitochondrial diseases remains unclear, and neuroimaging has not revealed specific anatomic patterns. A hypothalamus-pituitary dysfunction has been suggested, focusing on mitochondrial function and neuronal activity involving glutamate transport: the GnRH pulse generator is inhibited by GABA and stimulated by neurotransmitters such as glutamate; impairment of GABAergic suppression may prematurely activate the GnRH pulse generator, causing PP. Environmental (diet, exercise habit, endocrine-disrupting chemicals, etc.) and disease-specific factors likely contribute as well (133).

In a study by Chae et al. (133), central PP was identified in 10 out of 140 female patients with mitochondrial diseases. All presented with advanced bone age and elevated LH levels on GnRH stimulation testing, and all had normal brain MRI (133). These findings indicate the need for monitoring pubertal development in children with mitochondrial disorders and referring those with signs of PP to a pediatric endocrinologist. In such cases, a GnRH stimulation test, brain MRI to exclude hypothalamus-pituitary lesions, and therapy with GnRH agonists are important (133).

Central hypothyroidism has also been described, including a 12-year-old girl with MELAS and severe neurological impairment with brain damage on MRI (3, 134).

Involvement of the GH/IGF1 axis is well documented in mitochondrial diseases. In fact, growth retardation is common in these patients, who usually present with short stature and lower body mass index (3, 134, 135). Hypothalamus-pituitary dysfunction has been proposed to explain GH deficiency, but other hypotheses include chronic ischemia and energy deficiency in the diencephalon due to mitochondrial abnormalities, as well as non-specific degenerative changes and cerebral atrophy (3). Delayed or absent puberty is also frequent in patients with short stature, further supporting possible hypothalamic-pituitary dysfunction (3). Patients with confirmed GH deficiency generally respond well to GH therapy, with rapid improvement and few or no adverse effects (3, 136–138).

Patients with hemochromatosis may exhibit hypogonadotropic hypogonadism, primarily attributed to pituitary iron deposition (139, 140). This manifestation is more extensively documented in males, who present with thinning body hair, loss of libido and impotence (141–147). In females, although more rarely described, it may manifest with dysmenorrhea, secondary amenorrhea, reduced fertility, early menopause and decreased libido (147, 148). Early phlebotomy, together with testosterone replacement therapy in males and estrogen in females, appears beneficial in restoring gonadal function (139, 149–152).Despite increasing recognition of hypothalamus-pituitary involvement in IMDs, significant uncertainties remain regarding the precise mechanisms, optimal monitoring strategies, and standardized treatment approaches, highlighting the need for further longitudinal studies and tailored clinical guidelines.

## 4 Conclusion

Metabolic diseases are multi-organ pathologies that frequently involve the endocrine system. An underlying inherited metabolic disorder should be considered when multiple endocrine abnormalities coexist or when an endocrine condition presents alongside other clinical features not directly related to it.

The pathogenesis of endocrine manifestations in these contexts often remains unclear, highlighting the need for further studies to elucidate the biochemical and biological mechanisms underlying these interactions. A deeper understanding could also guide the development of more targeted therapies.

In certain cases, endocrine disorders secondary to a metabolic disease may respond to specific treatments distinct from the therapy for the primary metabolic condition. Therefore, early recognition of an underlying metabolic disorder can be crucial for improving patient outcomes, particularly in complex cases with endocrine involvement.

**Figure 3:**
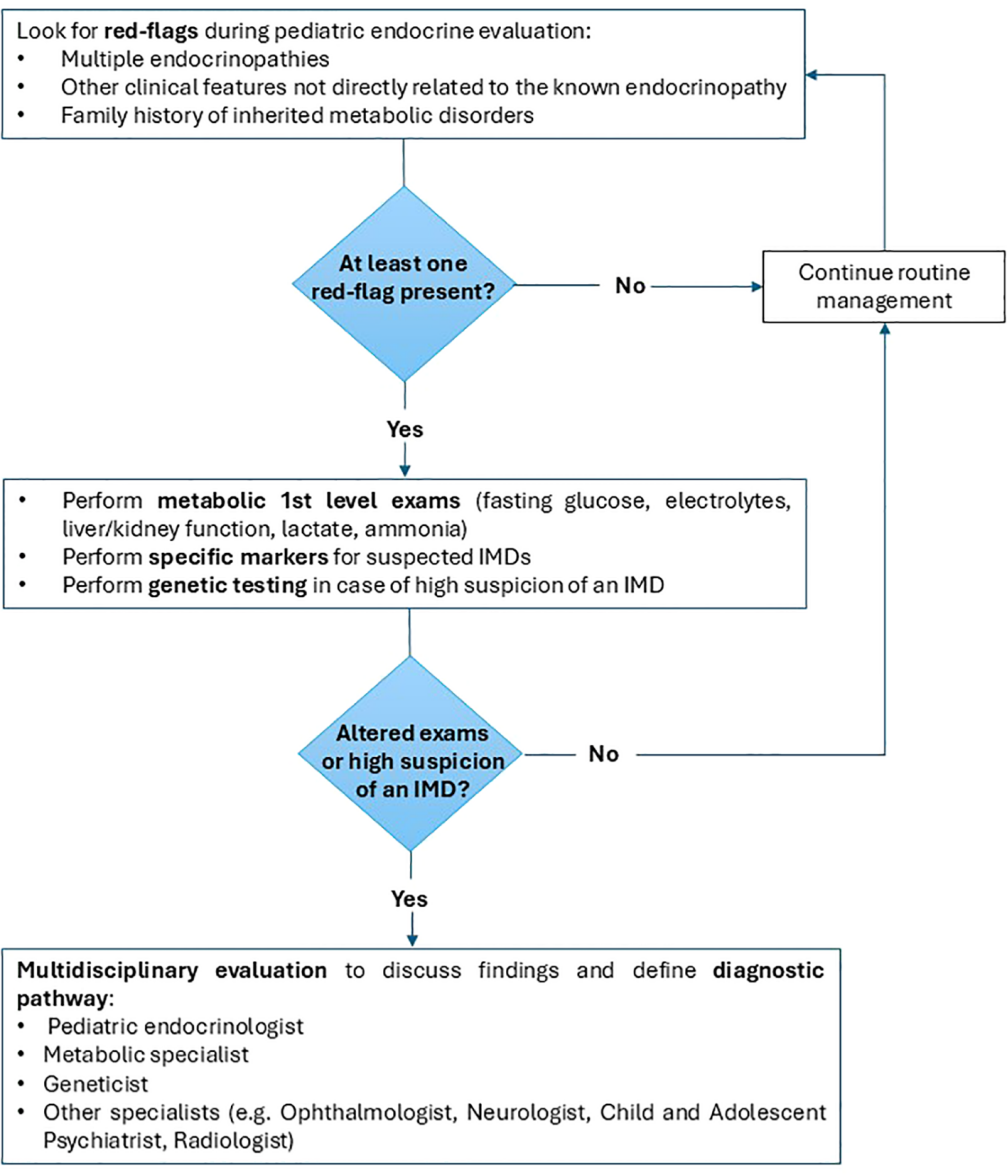
Proposed diagnostic algorithm for recognizing inherited metabolic diseases in pediatric endocrine practice.

Despite evidence of endocrine organ or system involvement in many of the inherited metabolic diseases analyzed, clear guidelines are lacking regarding the timing of follow-up or the initiation of specific hormonal therapies. Multidisciplinary discussion is essential to develop precise recommendations tailored to each endocrine complication across the various IMDs. To address this gap, we propose a summarized clinical diagnostic and management algorithm for recognizing inherited metabolic disorders in pediatric endocrine practice (**Figure 3**). Implementation of such an algorithm could facilitate early diagnosis, optimize endocrine care, and ultimately improve outcomes in pediatric patients with IMDs.

## References

[1] Blau N Dionisi Vici C Ferreira CR Vianey-Saban C Van Karnebeek CDM . Physician’s Guide to the Diagnosis, Treatment, and Follow-Up of Inherited Metabolic Diseases. Cham: Springer International Publishing (2022). doi: 10.1007/978-3-030-67727-5

[2] Saudubray JM Baumgartner MR Walter J . Inborn Metabolic Diseases. Berlin, Heidelberg: Springer Berlin Heidelberg (2016). doi: 10.1007/978-3-662-49771-5

[3] Chow J Rahman J Achermann JC Dattani MT Rahman S . Mitochondrial disease and endocrine dysfunction. Nat Rev Endocrinol. (2017) 13:92–104. doi: 10.1038/nrendo.2016.151, PMID: 27716753

[4] Varughese R Rahman S . Endocrine dysfunction in primary mitochondrial diseases. Endocrine Rev. (2025) 46:376–96. doi: 10.1210/endrev/bnaf002, PMID: 39891580 PMC12063101

[5] Bacon BR Adams PC Kowdley KV Powell LW Tavill AS . Diagnosis and management of hemochromatosis: 2011 Practice Guideline by the American Association for the Study of Liver Diseases. Hepatology. (2011) 54:328–43. doi: 10.1002/hep.24330, PMID: 21452290 PMC3149125

[6] Spiegel R Rakover-Tenenbaum Y Mandel H Lumelski D Admoni O Horovitz Y . Secondary diabetes mellitus: late complication of glycogen storage disease type 1b. J Pediatr Endocrinol Metab. (2005) 18. doi: 10.1515/JPEM.2005.18.6.617/html, PMID: 16042332

[7] Ismail H . Glycogen storage disease type III presenting with secondary diabetes and managed with insulin: a case report. cases J. (2009). doi: 10.4076/1757-1627-2-6891, PMID: 19829878 PMC2740253

[8] Salih M . Inborn errors of metabolism associated with hyperglycaemic ketoacidosis and diabetes mellitus: narrative review. Sudan J Paed. (2018) 18:10–23. doi: 10.24911/SJP.2018.1.3, PMID: 30166758 PMC6113785

[9] Faggiano A Pisani A Milone F Gaccione M Filippella M Santoro A . Endocrine dysfunction in patients with fabry disease. J Clin Endocrinol Metab. (2006) 91:4319–25. doi: 10.1210/jc.2006-0858, PMID: 16926253

[10] Bothou C Beuschlein F Nowak A . Endocrine disorders in patients with Fabry disease: insights from a reference centre prospective study. Endocrine. (2022) 75:728–39. doi: 10.1007/s12020-021-02918-4, PMID: 34751898 PMC8888367

[11] Faggiano A Severino R Ramundo V Russo R Vuolo L Del Prete M . Thyroid function in Fabry disease before and after enzyme replacement therapy. Minerva Endocrinol. (2011) 36:1–5., PMID: 21460782

[12] Lucky AW Howley PM Megyesi K Spielberg SP Schulman JD . Endocrine studies in cystinosis: Compensated primary hypothyroidism. J Pediatrics. (1977) 91:204–10. doi: 10.1016/S0022-3476(77)80813-5, PMID: 406375

[13] Das G Nanda PM Kaur A Kumar R . Bartter syndrome and hypothyroidism masquerading cystinosis in a 3-year-old girl: rare manifestation of a rare disease. BMJ Case Rep. (2021) 14:e242954. doi: 10.1136/bcr-2021-242954, PMID: 34312133 PMC8314690

[14] Elmonem MA Veys KR Soliman NA Van Dyck M Van Den Heuvel LP Levtchenko E . Cystinosis: a review. Orphanet J Rare Dis. (2016) 11:47. doi: 10.1186/s13023-016-0426-y, PMID: 27102039 PMC4841061

[15] Emma F Nesterova G Langman C Labbe A Cherqui S Goodyer P . Nephropathic cystinosis: an international consensus document. Nephrol Dialysis Transplant. (2014) 29:iv87–94. doi: 10.1093/ndt/gfu090, PMID: 25165189 PMC4158338

[16] Burke JR El-Bishti MM Maisey MN Chantler C . Hypothyroidism in children with cystinosis. Arch Dis Childhood. (1978) 53:947–51. doi: 10.1136/adc.53.12.947, PMID: 747399 PMC1545156

[17] Ünsal Y Özön ZA . Endocrine implications of congenital disorders of glycosylation. Jcrpe. (2025). doi: 10.4274/jcrpe.galenos.2025.2024-10-7, PMID: 39975416 PMC12989901

[18] Mohamed M Theodore M van DGHC AE VH Huijben K Van Dongen L . Thyroid function in PMM2-CDG: Diagnostic approach and proposed management. Mol Genet Metab. (2012) 105:681–3. doi: 10.1016/j.ymgme.2012.02.001, PMID: 22386715

[19] Macchia PE Harrison HH Scherberg NH Sunthornthepfvarakul T Jaeken J Refetoff S . Thyroid function tests and characterization of thyroxine-binding globulin in the carbohydrate-deficient glycoprotein syndrome type I. J Clin Endocrinol Metab. (1995) 80:3744–9. doi: 10.1210/jcem.80.12.8530628, PMID: 8530628

[20] Alsharhan H Ng BG Daniel EJP Friedman J Pivnick EK Al-Hashem A . Expanding the phenotype, genotype and biochemical knowledge of ALG3-CDG. J Inher Metab Disea. (2021) 44:987–1000. doi: 10.1002/jimd.12367, PMID: 33583022 PMC8282734

[21] Broomfield A Sweeney MG Woodward CE Fratter C Morris AM Leonard JV . Paediatric single mitochondrial DNA deletion disorders: an overlapping spectrum of disease. J Inher Metab Disea. (2015) 38:445–57. doi: 10.1007/s10545-014-9778-4, PMID: 25352051 PMC4432108

[22] Harvey JN Barnett D . Endocrine dysfunction in Kearns-Sayre syndrome. Clin Endocrinology. (1992) 37:97–104. doi: 10.1111/j.1365-2265.1992.tb02289.x, PMID: 1424198

[23] Berio A Piazzi A . Multiple endocrinopathies (growth hormone deficiency, autoimmune hypothyroidism and diabetes mellitus) in Kearns-Sayre syndrome. Pediatr Med Chir. (2013) 35. Available online at: http://pediatrmedchir.org/index.php/pmc/article/view/48 (Accessed March 26, 2025)., PMID: 23947115 10.4081/pmc.2013.48

[24] Hopkins SE Somoza A Gilbert DL . Rare autosomal dominant POLG1 mutation in a family with metabolic strokes, posterior column spinal degeneration, and multi-endocrine disease. J Child Neurol. (2010) 25:752–6. doi: 10.1177/0883073809343313, PMID: 19815814

[25] Emma F Bertini E Salviati L Montini G . Renal involvement in mitochondrial cytopathies. Pediatr Nephrol. (2012) 27:539–50. doi: 10.1007/s00467-011-1926-6, PMID: 21656172 PMC3288375

[26] Lee YS Yap HK Barshop BA Lee YS Rajalingam S Loke KY . Mitochondrial tubulopathy: the many faces of mitochondrial disorders. Pediatr Nephrology. (2001) 16:710–2. doi: 10.1007/s004670100637, PMID: 11511982

[27] Katsanos KH Elisaf M Bairaktari E Tsianos EV . Severe hypomagnesemia and hypoparathyroidism in kearns-sayre syndrome. Am J Nephrol. (2001) 21:150–3. doi: 10.1159/000046239, PMID: 11359024

[28] Ala A Walker AP Ashkan K Dooley JS Schilsky ML . Wilson’s disease. Lancet. (2007) 369:397–408. doi: 10.1016/S0140-6736(07)60196-2, PMID: 17276780

[29] Dzieżyc K Litwin T Członkowska A . Other organ involvement and clinical aspects of Wilson disease. In: Handbook of Clinical Neurology. Amsterdam, Netherlands: Elsevier (2017). p. 157–69. Available online at: https://linkinghub.elsevier.com/retrieve/pii/B9780444636256000136 (Accessed March 26, 2025)., PMID: 10.1016/B978-0-444-63625-6.00013-6 28433099

[30] Carpenter TO Carnes DL Anast CS . Hypoparathyroidism in wilson’s disease. N Engl J Med. (1983) 309:873–7. doi: 10.1056/NEJM198310133091501, PMID: 6888480

[31] Sawant R Chaudhari P Hamdulay KF Kumar S Acharya S . Beyond the norm: unusual coexistence of wilson disease and hypoparathyroidism. Cureus. (2024). doi: 10.7759/cureus.54516, PMID: 38516426 PMC10955449

[32] Fatima J Karoli R Jain V . Hypoparathyroidism in a case of Wilson′s disease: Rare association of a rare disorder. Indian J Endocr Metab. (2013) 17:361. doi: 10.4103/2230-8210.109689, PMID: 23776928 PMC3683230

[33] Dionisi-Vici C Garavaglia B Burlina AB Bertini E Saponara I Sabetta G . Hypoparathyroidism in mitochondrial trifunctional protein deficiency. J Pediatrics. (1996) 129:159–62. doi: 10.1016/s0022-3476(96)70206-8, PMID: 8757579

[34] Naiki M Ochi N Kato YS Purevsuren J Yamada K Kimura R . Mutations in *HADHB*, which encodes the β-subunit of mitochondrial trifunctional protein, cause infantile onset hypoparathyroidism and peripheral polyneuropathy. Am J Med Genet Pt A. (2014) 164:1180–7. doi: 10.1002/ajmg.a.36434, PMID: 24664533

[35] Labarthe F Benoist JF Brivet M Vianey-Saban C Despert F . Ogier De Baulny H. Partial hypoparathyroidism associated with mitochondrial trifunctional protein deficiency. Eur J Pediatr. (2006) 165:389–91. doi: 10.1007/s00431-005-0052-5, PMID: 16523289

[36] Tyni T Rapola J Palotie A Pihko H . Hypoparathyroidism in a patient with long-chain 3-hydroxyacyl-coenzyme A dehydrogenase deficiency caused by the G1528C mutation. J Pediatrics. (1997) 131:766–8. doi: 10.1016/s0022-3476(97)70111-2, PMID: 9403664

[37] Saudubray JM Martin D De Lonlay P Touati G Poggi-Travert F Bonnet D . Recognition and management of fatty acid oxidation defects: A series of 107 patients. J Inher Metab Disea. (1999) 22:487–502. doi: 10.1023/a:1005556207210, PMID: 10407781

[38] Moser H . Adrenoleukodystrophy: phenotype, genetics, pathogenesis and therapy. Brain. (1997) 120:1485–508. doi: 10.1093/brain/120.8.1485, PMID: 9278636

[39] Kanakis G Kaltsas G . Adrenal Insufficiency Due to X-Linked Adrenoleukodystrophy. In: Feingold KR Anawalt B Blackman MR Boyce A Chrousos G Corpas E , editors. Endotext [Internet]. South Dartmouth Massachusetts (MA): MDText.com, Inc (2000). Available online at: http://www.ncbi.nlm.nih.gov/books/NBK278944/.

[40] Assies J Gooren LJG Geel BV Barth PG . Signs of testicular insufficiency in adrenomyeloneuropathy and neurologically asymptomatic X-linked adrenoleukodystrophy: a retrospective study. Int J Andrology. (1997) 20:315–21. doi: 10.1046/j.1365-2605.1997.00066.x, PMID: 16130276

[41] Karapanou O Vlassopoulou B Tzanela M Papadopoulos D Angelidakis P Michelakakis H . X-linked adrenoleukodystrophy: are signs of hypogonadism always due to testicular failure? Hormones. (2014) 13:146–52. doi: 10.1007/BF03401330, PMID: 24722136

[42] Stradomska TJ Kubalska J Janas R Tylki-Szymańska A . Reproductive function in men affected by X-linked adrenoleukodystrophy/adrenomyeloneuropathy. Eur J Endocrinology. (2012) 166:291–4. doi: 10.1530/EJE-11-0490, PMID: 22048970

[43] Aversa A Palleschi S Cruccu G Silvestroni L Isidori A Fabbri A . Rapid decline of fertility in a case of adrenoleukodystrophy. Hum Reproduction. (1998) 13:2474–9. doi: 10.1093/humrep/13.9.2474, PMID: 9806270

[44] Fridovich-Keil JL Gubbels CS Spencer JB Sanders RD Land JA Rubio-Gozalbo E . Ovarian function in girls and women with GALT-deficiency galactosemia. J Inher Metab Disea. (2011) 34:357–66. doi: 10.1007/s10545-010-9221-4, PMID: 20978943 PMC3063539

[45] Berry GT Walter J Fridovich-Keil JL . Disorders of Galactose Metabolism. In: Saudubray JM Baumgartner MR Walter J , editors. Inborn Metabolic Diseases. Springer Berlin Heidelberg, Berlin, Heidelberg (2016). p. 139–47. doi: 10.1007/978-3-662-49771-5_6

[46] Badik JR Castañeda U Gleason TJ Spencer JB Epstein MP Ficicioglu C . Ovarian function in Duarte galactosemia. Fertility Sterility. (2011) 96:469–73. doi: 10.1016/j.fertnstert.2011.05.088, PMID: 21719007 PMC3773175

[47] Welling L Bernstein LE Berry GT Burlina AB Eyskens F Gautschi M . International clinical guideline for the management of classical galactosemia: diagnosis, treatment, and follow-up. J Inher Metab Disea. (2017) 40:171–6. doi: 10.1007/s10545-016-9990-5, PMID: 27858262 PMC5306419

[48] Van Erven B Gubbels CS Van Golde RJ Dunselman GA Derhaag JG De Wert G . Fertility preservation in female classic galactosemia patients. Orphanet J Rare Dis. (2013) 8:107. doi: 10.1186/1750-1172-8-107, PMID: 23866841 PMC3718676

[49] Rubio-Gozalbo ME Panis B Zimmermann LJI Spaapen LJ Menheere PPCA . The endocrine system in treated patients with classical galactosemia. Mol Genet Metab. (2006) 89:316–22. doi: 10.1016/j.ymgme.2006.07.005, PMID: 16935538

[50] Liu G . Galactose metabolism and ovarian toxicity. Reprod Toxicology. (2000) 14:377–84. doi: 10.1016/s0890-6238(00)00096-4, PMID: 11020650

[51] Ronin C . Glycosylation of pituitary hormones: a necessary and multistep control of biopotency. Glycoconj J. (1992) 9:279–83. doi: 10.1007/BF00731085, PMID: 1305419

[52] Akintayo A Stanley P . Roles for golgi glycans in oogenesis and spermatogenesis. Front Cell Dev Biol. (2019) 7:98. doi: 10.3389/fcell.2019.00098, PMID: 31231650 PMC6566014

[53] Langman CB Delos Santos RB Ghossein C Atherton AM Levtchenko EN Servais A . Fertility management in cystinosis: A clinical perspective. Kidney Int Rep. (2024) 9:214–24. doi: 10.1016/j.ekir.2023.10.030, PMID: 38344731 PMC10851017

[54] Reda A Veys K Besouw M . Fertility in cystinosis. Cells. (2021) 10:3539. doi: 10.3390/cells10123539, PMID: 34944047 PMC8700558

[55] Niaudet P . Cystinosis. In: Saudubray JM Baumgartner MR Walter J , editors. Inborn Metabolic Diseases. Springer Berlin Heidelberg, Berlin, Heidelberg (2016). p. 623–9. doi: 10.1007/978-3-662-49771-5_42

[56] Hauser AC Gessl A Harm F Wiesholzer M Kleinert J Wallner M . Hormonal profile and fertility in patients with Anderson-Fabry disease: Hormones and Anderson-Fabry Disease. Int J Clin Practice. (2005) 59:1025–8. doi: 10.1111/j.1742-1241.2005.00620.x, PMID: 16115176

[57] Nistal M Paniagua R Picazo ML . Testicular and epididymal involvement in Fabry’s disease. J Pathology. (1983) 141:113–24. doi: 10.1002/path.1711410203, PMID: 6420529

[58] Chakraborty J Hikim APS Jhunjhunwala JS . Stagnation of blood in the microcirculatory vessels in the testes of men with varicocele. J Andrology. (1985) 6:117–26. doi: 10.1002/j.1939-4640.1985.tb00826.x, PMID: 3988623

[59] Germain DP Altarescu G Barriales-Villa R Mignani R Pawlaczyk K Pieruzzi F . An expert consensus on practical clinical recommendations and guidance for patients with classic Fabry disease. Mol Genet Metab. (2022) 137:49–61. doi: 10.1016/j.ymgme.2022.07.010, PMID: 35926321

[60] Kishnani PS Austin SL Abdenur JE Arn P Bali DS Boney A . Diagnosis and management of glycogen storage disease type I: a practice guideline of the American College of Medical Genetics and Genomics. Genet Med. (2014) 16:e1–29. doi: 10.1038/gim.2014.128, PMID: 25356975

[61] Sechi A Deroma L Lapolla A Paci S Melis D Burlina A . Fertility and pregnancy in women affected by glycogen storage disease type I, results of a multicenter Italian study. J Inher Metab Disea. (2013) 36:83–9. doi: 10.1007/s10545-012-9490-1, PMID: 22562700

[62] Bali DS El-Gharbawy A Austin S Pendyal S Kishnani PS . Glycogen Storage Disease Type I. In: Adam MP Bick S Mirzaa GM Pagon RA Wallace SE Amemiya A , editors. GeneReviews^®^. University of Washington, Seattle, Seattle (WA (2006). p. 1993–2024. 20301489

[63] Lee PJ Patel A Hindmarsh PC Mowat AP Leonard JV . The prevalence of polycystic ovaries in the hepatic glycogen storage diseases: its association with hyperinsulinism. Clin Endocrinology. (1995) 42:601–6. doi: 10.1111/j.1365-2265.1995.tb02686.x, PMID: 7634500

[64] Austin SL El-Gharbawy AH Kasturi VG James A Kishnani PS . Menorrhagia in patients with type I glycogen storage disease. Obstetrics Gynecology. (2013) 122:1246–54. doi: 10.1097/01.AOG.0000435451.86108.82, PMID: 24201678

[65] Edlund M Blombäck M Fried G . Desmopressin in the treatment of menorrhagia in women with no common coagulation factor deficiency but with prolonged bleeding time. Blood Coagulation Fibrinolysis. (2002) 13:225–31. doi: 10.1097/00001721-200204000-00008, PMID: 11943936

[66] Marti GE Rick ME Sidbury J Gralnick HR . DDAVP infusion in five patients with type Ia glycogen storage disease and associated correction of prolonged bleeding times. Blood. (1986) 68:180–4. doi: 10.1182/blood.V68.1.180.180, PMID: 3087438

[67] Mairovitz V Labrune P Fernandez H Audibert F Frydman R . Contraception and pregnancy in women affected by glycogen storage diseases. Eur J Pediatrics. (2002) 161:S97–101. doi: 10.1007/s00431-002-1013-x, PMID: 12373581

[68] Bahamondes L Monteiro-Dantas C Espejo-Arce X Dos Santos Fernandes AM Lui-Filho JF Perrotti M . A prospective study of the forearm bone density of users of etonorgestrel- and levonorgestrel-releasing contraceptive implants. Hum Reproduction. (2006) 21:466–70. doi: 10.1093/humrep/dei358, PMID: 16253974

[69] Bakhshalizadeh S Hock DH Siddall NA Kline BL Sreenivasan R Bell KM . Deficiency of the mitochondrial ribosomal subunit, MRPL50, causes autosomal recessive syndromic premature ovarian insufficiency. Hum Genet. (2023) 142:879–907. doi: 10.1007/s00439-023-02563-z, PMID: 37148394 PMC10329598

[70] Rahman S Mayr JA . Disorders of Oxidative Phosphorylation. In: Saudubray JM Baumgartner MR Walter J , editors. Inborn Metabolic Diseases. Springer Berlin Heidelberg, Berlin, Heidelberg (2016). p. 223–42. doi: 10.1007/978-3-662-49771-5_14

[71] Zhang ZL Ren ST Yang WJ Xu XW Zhao SM Fang KF . AARS2-catalyzed lactylation induces follicle development and premature ovarian insufficiency. Cell Death Discov. (2025) 11:209. doi: 10.1038/s41420-025-02501-0, PMID: 40301335 PMC12041370

[72] Kemp S Huffnagel IC Linthorst GE Wanders RJ Engelen M . Adrenoleukodystrophy – neuroendocrine pathogenesis and redefinition of natural history. Nat Rev Endocrinol. (2016) 12:606–15. doi: 10.1038/nrendo.2016.90, PMID: 27312864

[73] Huffnagel IC Laheji FK Aziz-Bose R Tritos NA Marino R Linthorst GE . The natural history of adrenal insufficiency in X-linked adrenoleukodystrophy: an international collaboration. J Clin Endocrinol Metab. (2019) 104:118–26. doi: 10.1210/jc.2018-01307, PMID: 30252065

[74] Ramirez Alcantara J Grant NR Sethuram S Nagy A Becker C Sahai I . Early detection of adrenal insufficiency: the impact of newborn screening for adrenoleukodystrophy. J Clin Endocrinol Metab. (2023) 108:e1306–15. doi: 10.1210/clinem/dgad286, PMID: 37220095 PMC11009790

[75] Dubey P Raymond GV Moser AB Kharkar S Bezman L Moser HW . Adrenal insufficiency in asymptomatic adrenoleukodystrophy patients identified by very long-chain fatty acid screening. J Pediatrics. (2005) 146:528–32. doi: 10.1016/j.jpeds.2004.10.067, PMID: 15812458

[76] Regelmann MO Kamboj MK Miller BS Nakamoto JM Sarafoglou K Shah S . Adrenoleukodystrophy: guidance for adrenal surveillance in males identified by newborn screen. J Clin Endocrinol Metab. (2018) 103:4324–31. doi: 10.1210/jc.2018-00920, PMID: 30289543

[77] Erdöl Ş Sağlam H . Endocrine dysfunctions in patients with inherited metabolic diseases. Jcrpe. (2016) 8:330–3. doi: 10.4274/jcrpe.2288, PMID: 27086477 PMC5096498

[78] Alsaleem M Saadeh L . Adrenoleukodystrophy. In: StatPearls. StatPearls Publishing, Treasure Island (FL (2024). Available online at: http://www.ncbi.nlm.nih.gov/books/NBK562328/., PMID:

[79] Vogel BH Bradley SE Adams DJ D’Aco K Erbe RW Fong C . Newborn screening for X-linked adrenoleukodystrophy in New York State: Diagnostic protocol, surveillance protocol and treatment guidelines. Mol Genet Metab. (2015) 114:599–603. doi: 10.1016/j.ymgme.2015.02.002, PMID: 25724074

[80] Andersson HC Frentz J Martínez JE Tuck-Muller CM Bellizaire J . Adrenal insufficiency in Smith-Lemli-Opitz syndrome. Am J Med Genet. (1999) 82:382–4. doi: 10.1002/(SICI)1096-8628(19990219)82:5<382::AID-AJMG5>3.0.CO;2-9, PMID: 10069708

[81] Chemaitilly W Goldenberg A Baujat G Thibaud E Cormier-Daire V Abadie V . Adrenal insufficiency and abnormal genitalia in a 46XX female with smith-lemli-opitz syndrome. Horm Res Paediatr. (2003) 59:254–6. doi: 10.1159/000070226, PMID: 12714790

[82] Nowaczyk MJM Siu VM Krakowiak PA Porter FD . Adrenal insufficiency and hypertension in a newborn infant with Smith-Lemli-Opitz syndrome. Am J Med Genet. (2001) 103:223–5. doi: 10.1002/ajmg.1545, PMID: 11745994

[83] Bianconi SE Conley SK Keil MF Sinaii N Rother KI Porter FD . Adrenal function in Smith–Lemli–Opitz syndrome. Am J Med Genet Pt A. (2011) 155:2732–8. doi: 10.1002/ajmg.a.34271, PMID: 21990131 PMC3488380

[84] Worthington S Goldblatt J . Smith-Lemli-Opitz syndrome: further delineation of the phenotype. Clin Dysmorphology. (1997) 6:263–6. doi: 10.1097/00019605-199707000-00011, PMID: 9220198

[85] Shackleton C Roitman E Guo LW Wilson WK Porter FD . Identification of 7(8) and 8(9) unsaturated adrenal steroid metabolites produced by patients with 7-dehydrosterol-Δ7-reductase deficiency (Smith–Lemli–Opitz syndrome). J Steroid Biochem Mol Biol. (2002) 82:225–32. doi: 10.1016/S0960-0760(02)00155-3, PMID: 12477489

[86] Irons M . Smith-Lemli-Oitz Syndrome. In: Pagan RA Bird TC Dolan CR Stephens K , editors. Gene Reviews. Univeristy of Washington, Seattle, Seattle (WA (1998). p. 1993. 20301322

[87] Waterham HR Clayton PT . Disorders of Isoprenoid/Cholesterol Synthesis. In: Saudubray JM Baumgartner MR Walter J , editors. Inborn Metabolic Diseases. Springer Berlin Heidelberg, Berlin, Heidelberg (2016). p. 455–64. doi: 10.1007/978-3-662-49771-5_32

[88] Afroze B Amjad N Ibrahim SH Humayun KN Yakob Y . Adrenal insufficiency in a child with MELAS syndrome. Brain Dev. (2014) 36:924–7. doi: 10.1016/j.braindev.2013.12.009, PMID: 24508408

[89] Corkery-Hayward M Metherell LA . Adrenal dysfunction in mitochondrial diseases. IJMS. (2023) 24:1126. doi: 10.3390/ijms24021126, PMID: 36674647 PMC9862368

[90] Artuch R Pavía C Playán A Vilaseca MA Colomer J Valls C . Multiple endocrine involvement in two pediatric patients with kearns-sayre syndrome. Horm Res Paediatr. (1998) 50:99–104. doi: 10.1159/000023243, PMID: 9701704

[91] Boles RG Roe T Senadheera D Mahnovski V Wong LJC . Mitochondrial DNA deletion with Kearns Sayre syndrome in a child with Addison disease. Eur J Pediatrics. (1998) 157:643–7. doi: 10.1007/s004310050902, PMID: 9727847

[92] Tzoufi M Makis A Chaliasos N Nakou I Siomou E Tsatsoulis A . A rare case report of simultaneous presentation of myopathy, Addison’s disease, primary hypoparathyroidism, and Fanconi syndrome in a child diagnosed with Kearns–Sayre syndrome. Eur J Pediatr. (2013) 172:557–61. doi: 10.1007/s00431-012-1798-1, PMID: 22875312

[93] Duran GP Martinez-Aguayo A Poggi H Lagos M Gutierrez D Harris PR . Large Mitochondrial DNA Deletion in an Infant with Addison Disease. In: JIMD Reports - Case and Research Reports. Springer Berlin Heidelberg, Berlin, Heidelberg (2011). p. 5–9. doi: 10.1007/8904_2011_33, PMID: PMC3509852 23430867

[94] Čechová A Honzík T Edmondson AC Ficicioglu C Serrano M Barone R . Should patients with Phosphomannomutase 2-CDG (PMM2-CDG) be screened for adrenal insufficiency? Mol Genet Metab. (2021) 133:397–9. doi: 10.1016/j.ymgme.2021.06.003, PMID: 34140212 PMC8754259

[95] Chabre O Goichot B Zenaty D Bertherat J . Group 1. Epidemiology of primary and secondary adrenal insufficiency: Prevalence and incidence, acute adrenal insufficiency, long-term morbidity and mortality. Annales d’Endocrinologie. (2017) 78:490–4. doi: 10.1016/j.ando.2017.10.010, PMID: 29174931

[96] Sjarif DR Ploos Van Amstel JK Duran M Beemer FA Poll-The BT . Isolated and contiguous glycerol kinase gene disorders: A review. J Inher Metab Disea. (2000) 23:529–47. doi: 10.1023/a:1005660826652, PMID: 11032329

[97] Wamelink MMC Valayannopoulos V Garavaglia B . Disorders of Glycolysis and the Pentose Phosphate Pathway. In: Saudubray JM Baumgartner MR Walter J , editors. Inborn Metabolic Diseases. Springer Berlin Heidelberg, Berlin, Heidelberg (2016). p. 149–60. doi: 10.1007/978-3-662-49771-5_7

[98] Korkut S Baştuğ O Raygada M Hatipoğlu N Kurtoğlu S Kendirci M . Complex glycerol kinase deficiency and adrenocortical insufficiency in two neonates. Jcrpe. (2016) 8:468–71. doi: 10.4274/jcrpe.2539, PMID: 27087023 PMC5198007

[99] Berendse K Engelen M Linthorst GE Van Trotsenburg AP Poll-The BT . High prevalence of primary adrenal insufficiency in Zellweger spectrum disorders. Orphanet J Rare Dis. (2014) 9:133. doi: 10.1186/s13023-014-0133-5, PMID: 25179809 PMC4164755

[100] Barton JC Acton RT . Diabetes in *HFE* hemochromatosis. J Diabetes Res. (2017) 2017:1–16. doi: 10.1155/2017/9826930, PMID: 28331855 PMC5346371

[101] Vantyghem MC Dobbelaere D Mention K Wemeau JL Saudubray JM Douillard C . Endocrine manifestations related to inherited metabolic diseases in adults. Orphanet J Rare Dis. (2012) 7:11. doi: 10.1186/1750-1172-7-11, PMID: 22284844 PMC3349544

[102] Fasano A Colosimo C Miyajima H Tonali PA Re TJ Bentivoglio AR . Aceruloplasminemia: A novel mutation in a family with marked phenotypic variability. Movement Disord. (2008) 23:751–5. doi: 10.1002/mds.21938, PMID: 18200628

[103] Guven A Cebeci N Dursun A Aktekin E Baumgartner M Fowler B . Methylmalonic acidemia mimicking diabetic ketoacidosis in an infant. Pediatr Diabetes. (2012) 13:e22–5. doi: 10.1111/j.1399-5448.2011.00784.x, PMID: 21545677

[104] Dejkhamron P Wejapikul K Unachak K Sawangareetrakul P Tanpaiboon P Wattanasirichaigoon D . Isolated methylmalonic acidemia with unusual presentation mimicking diabetic ketoacidosis. J Pediatr Endocrinol Metab. (2016) 29. doi: 10.1515/jpem-2015-0228/html, PMID: 26581066

[105] Baumgartner MR Hörster F Dionisi-Vici C Haliloglu G Karall D Chapman KA . Proposed guidelines for the diagnosis and management of methylmalonic and propionic acidemia. Orphanet J Rare Dis. (2014) 9:130. doi: 10.1186/s13023-014-0130-8, PMID: 25205257 PMC4180313

[106] Dweikat IM Naser EN Abu Libdeh AI Naser OJ Abu Gharbieh NN Maraqa NF . Propionic acidemia mimicking diabetic ketoacidosis. Brain Dev. (2011) 33:428–31. doi: 10.1016/j.braindev.2010.06.016, PMID: 20634010

[107] Joshi R Phatarpekar A . Propionic acidemia presenting as diabetic ketoacidosis. Indian Pediatr. (2011) 48:164–5., PMID: 21378435

[108] Hou JW Wang TR . Isovaleric acidemia: report of one case. Zhonghua Min Guo Xiao Er Ke Yi Xue Hui Za Zhi. (1990) 31:262–5., PMID: 2124776

[109] Erdem E Cayonu N Uysalol E Yildirmak ZY . Chronic intermittent form of isovaleric acidemia mimicking diabetic ketoacidosis. J Pediatr Endocrinol Metab. (2010) 23. doi: 10.1515/jpem.2010.082/html, PMID: 20662350

[110] Kılıç M Kaymaz N Özgül RK . Isovaleric acidemia presenting as diabetic ketoacidosis: A case report. Jcrpe. (2014) 6:59–61. doi: 10.4274/Jcrpe.1181, PMID: 24637313 PMC3986742

[111] Hou JW . Biotin responsive multiple carboxylase deficiency presenting as diabetic ketoacidosis. Chang Gung Med J. (2004) 27:129–33., PMID: 15095958

[112] Hwang WJ Lim HH Kim YM Chang MY Kil HR Kim JY . Pancreatic involvement in patients with inborn errors of metabolism. Orphanet J Rare Dis. (2021) 16:37. doi: 10.1186/s13023-021-01685-9, PMID: 33472655 PMC7819202

[113] Filippi L Gozzini E Cavicchi C Morrone A Fiorini P Donzelli G . Insulin-resistant hyperglycaemia complicating neonatal onset of methylmalonic and propionic acidaemias. J Inher Metab Disea. (2009) 32:179–86. doi: 10.1007/s10545-009-1141-9, PMID: 19588269

[114] Imen M Hanene B Ichraf K Aida R Ilhem T Naziha K . Methylmalonic acidemia and hyperglycemia: An unusual association. Brain Dev. (2012) 34:113–4. doi: 10.1016/j.braindev.2011.07.002, PMID: 21802231

[115] Maassen JA T Hart L Van Essen E RJ H Nijpels G Jahangir Tafrechi RS . Mitochondrial diabetes. Diabetes. (2004) 53:S103–9. doi: 10.2337/diabetes.53.2007.S103, PMID: 14749274

[116] Whittaker RG Schaefer AM McFarland R Taylor RW Walker M Turnbull DM . Prevalence and progression of diabetes in mitochondrial disease. Diabetologia. (2007) 50:2085–9. doi: 10.1007/s00125-007-0779-9, PMID: 17653689

[117] Nesbitt V Pitceathly RDS Turnbull DM Taylor RW Sweeney MG Mudanohwo EE . The UK MRC Mitochondrial Disease Patient Cohort Study: clinical phenotypes associated with the m.3243A>G mutation–implications for diagnosis and management. J Neurology Neurosurg Psychiatry. (2013) 84:936–8. doi: 10.1136/jnnp-2012-303528, PMID: 23355809

[118] Karaa A Goldstein A . The spectrum of clinical presentation, diagnosis, and management of mitochondrial forms of diabetes: Diabetes in mitochondrial diseases. Pediatr Diabetes. (2015) 16:1–9. doi: 10.1111/pedi.12223, PMID: 25330715

[119] Schaefer AM Walker M Turnbull DM Taylor RW . Endocrine disorders in mitochondrial disease. Mol Cell Endocrinology. (2013) 379:2–11. doi: 10.1016/j.mce.2013.06.004, PMID: 23769710 PMC3820028

[120] Sharari S Abou-Alloul M Hussain K Ahmad Khan F . Fanconi–bickel syndrome: A review of the mechanisms that lead to dysglycaemia. IJMS. (2020) 21:6286. doi: 10.3390/ijms21176286, PMID: 32877990 PMC7504390

[121] Trepiccione F Iervolino A D’Acierno M Siccardi S Costanzo V Sardella D . The SGLT2 inhibitor dapagliflozin improves kidney function in glycogen storage disease XI. Sci Transl Med. (2023) 15:eabn4214. doi: 10.1126/scitranslmed.abn4214, PMID: 37910600

[122] Bouyahia O Ouderni M Ben Mansour F Matoussi N Khaldi F . Diabetic acido-ketosis revealing thiamine-responsive megaloblastic anemia. Annales d’Endocrinologie. (2009) 70:477–9. doi: 10.1016/j.ando.2009.09.001, PMID: 19922902

[123] Alzahrani AS Baitei E Zou M Shi Y . Thiamine transporter mutation: an example of monogenic diabetes mellitus. Eur J Endocrinol. (2006) 155:787–92. doi: 10.1530/eje.1.02305, PMID: 17132746

[124] Sako S Tsunogai T Oishi K . Thiamine-Responsive Megaloblastic Anemia Syndrome. In: Adam MP Feldman J Mirzaa GM Pagon RA Wallace SE Amemiya A , editors. GeneReviews^®^. University of Washington, Seattle, Seattle (WA (1993). Available online at: http://www.ncbi.nlm.nih.gov/books/NBK1282/., PMID: 20301459

[125] Bappal B Nair R Shaikh H AI Khusaiby SM de Silva V . Five years followup of diabetes mellitus in two siblings with thiamine responsive megaloblastic anemia. Indian Pediatr. (2001) 38:1295–8., PMID: 11721072

[126] Valerio G Franzese A Poggi V Tenore A . Long-term follow-up of diabetes in two patients with thiamine-responsive megaloblastic anemia syndrome. Diabetes Care. (1998) 21:38–41. doi: 10.2337/diacare.21.1.38, PMID: 9538968

[127] Gahl WA Balog JZ Kleta R . Nephropathic cystinosis in adults: natural history and effects of oral cysteamine therapy. Ann Intern Med. (2007) 147:242–50. doi: 10.7326/0003-4819-147-4-200708210-00006, PMID: 17709758

[128] Filler G Amendt P Von Bredow MA Ehrich JHH Rohde W . Slowly deteriorating insulin secretion and C-peptide production characterizes diabetes mellitus in infantile cystinosis. Eur J Pediatrics. (1998) 157:738–42., PMID: 9776533 10.1007/s004310050926

[129] Girard D Petrovsky N . Alström syndrome: insights into the pathogenesis of metabolic disorders. Nat Rev Endocrinol. (2011) 7:77–88. doi: 10.1038/nrendo.2010.210, PMID: 21135875

[130] Marshall JD Bronson RT Collin GB Nordstrom AD Maffei P Paisey RB . New alström syndrome phenotypes based on the evaluation of 182 cases. Arch Intern Med. (2005) 165:675. doi: 10.1001/archinte.165.6.675, PMID: 15795345

[131] Mokashi A Cummings EA . Presentation and course of diabetes in children and adolescents with Alstrom syndrome. Pediatr Diabetes. (2011) 12:270–5. doi: 10.1111/j.1399-5448.2010.00698.x, PMID: 21518413

[132] Moravej H Altassan R Jaeken J Enns GM Ellaway C Balasubramaniam S . Hypoglycemia in CDG patients due to PMM2 mutations: Follow up on hyperinsulinemic patients. JIMD Rep. (2020) 51:76–81. doi: 10.1002/jmd2.12085, PMID: 32071842 PMC7012739

[133] Chae HW Na JH Kwon A Kim HS Lee YM . Central precocious puberty may be a manifestation of endocrine dysfunction in pediatric patients with mitochondrial disease. Eur J Pediatr. (2021) 180:425–32. doi: 10.1007/s00431-020-03804-3, PMID: 32914201

[134] Balestri P Grosso S . Endocrine disorders in two sisters affected by MELAS syndrome. J Child Neurol. (2000) 15:755–8. doi: 10.1177/088307380001501108, PMID: 11108510

[135] Matsuzaki M Izumi T Shishikura K Suzuki H Hirayama Y . Hypothalamic growth hormone deficiency and supplementary GH therapy in two patients with mitochondrial myopathy, encephalopathy, lactic acidosis and stroke-like episodes. Neuropediatrics. (2002) 33:271–3. doi: 10.1055/s-2002-36742, PMID: 12536371

[136] Rocha V Rocha D Santos H Marques JS . Growth hormone deficiency in a patient with mitochondrial disease. J Pediatr Endocrinol Metab. (2015) 28. doi: 10.1515/jpem-2014-0315/html, PMID: 25781523

[137] Romano S Samara D Crosnier H Valayannopoulos V Polak M Chrétien D . Variable outcome of growth hormone administration in respiratory chain deficiency. Mol Genet Metab. (2008) 93:195–9. doi: 10.1016/j.ymgme.2007.09.007, PMID: 17951089

[138] Burns EC Preece MA Cameron N Tanner JM . GROWTH HORMONE DEFICIENCY IN MITOCHONDRIAL CYTOPATHY. Acta Paediatrica. (1982) 71:693–7. doi: 10.1111/j.1651-2227.1982.tb09504.x, PMID: 7136691

[139] Pelusi C Gasparini DI Bianchi N Pasquali R . Endocrine dysfunction in hereditary hemochromatosis. J Endocrinol Invest. (2016) 39:837–47. doi: 10.1007/s40618-016-0451-7, PMID: 26951056

[140] Bergeron C Kovacs K . Pituitary siderosis. A histologic, immunocytologic, and ultrastructural study. Am J Pathol. (1978) 93:295–309. doi: 10.1097/00005072-197809000-00068, PMID: 362939 PMC2018382

[141] McNeil LW McKee LC Lorber D Rabin D . The endocrine manifestations of hemochromatosis. Am J Med Sci. (1983) 285:7–13. doi: 10.1097/00000441-198305000-00002, PMID: 6342390

[142] Walsh CH Wright AD Holder G . A study of pituitary function in patients with idiopathic hemochromatosis. J Clin Endocrinol Metab. (1976) 43:866–72. doi: 10.1210/jcem-43-4-866, PMID: 824300

[143] Stocks AE Martin FIR . Pituitary function in haemochromatosis. Am J Med. (1968) 45:839–45. doi: 10.1016/0002-9343(68)90182-4, PMID: 4177545

[144] Charbonnel B Chupin M Le Grand A Guillon J . Pituitary function in idiopathic haemochromatosis: hormonal study in 36 male patients. Acta Endocrinologica. (1981) 98:178–83. doi: 10.1530/acta.0.0980178, PMID: 6794282

[145] Kley HK Stremmel W Niederau C Hehrmann R Shams O Strohmeyer G . Androgen and estrogen response to adrenal and gonadal stimulation in idiopathic hemochromatosis: evidence for decreased estrogen formation. Hepatology. (1985) 5:251–6. doi: 10.1002/hep.1840050216, PMID: 2984101

[146] Duranteau L Chanson P Blumberg-Tick J Thomas G Brailly S Lubetzki J . Non-responsiveness of serum gonadotropins and testosterone to pulsatile GnRH in hemochromatosis suggesting a pituitary defect. Acta Endocrinologica. (1993) 128:351–4. doi: 10.1530/acta.0.1280351, PMID: 8498154

[147] McDermott JH Walsh CH . Hypogonadism in hereditary hemochromatosis. J Clin Endocrinol Metab. (2005) 90:2451–5. doi: 10.1210/jc.2004-0980, PMID: 15657376

[148] Kelly TM Edwards CQ Meikle AW Kushner JP . Hypogonadism in hemochromatosis: reversal with iron depletion. Ann Intern Med. (1984) 101:629–32. doi: 10.7326/0003-4819-101-5-629, PMID: 6435491

[149] Farina G Pedrotti C Cerani P Rovati A Strada E Bergamaschi G . Successful pregnancy following gonadotropin therapy in a young female with juvenile idiopathic hemochromatosis and secondary hypogonadotropic hypogonadism. Haematologica. (1995) 80:335–7., PMID: 7590503

[150] Militaru MS Popp RA Trifa AP . Homozygous G320V mutation in the HJV gene causing juvenile hereditary haemochromatosis type A. A Case Rep J Gastrointestin Liver Dis. (2010) 19:191–3., PMID: 20593054

[151] Cundy T Butler J Bomford A Williams R . Reversibility of hypogonadotrophic hypogonadism associated with genetic haemochromatosis. Clin Endocrinology. (1993) 38:617–20. doi: 10.1111/j.1365-2265.1993.tb02143.x, PMID: 8334747

[152] Hamer OW Gnad M Schölmerich J Palitzsch KD . Successful treatment of erectile dysfunction and infertility by venesection in a patient with primary haemochromatosis. Eur J Gastroenterol Hepatol. (2001) 13:985–8. doi: 10.1097/00042737-200108000-00021, PMID: 11507369

[153] Chen CM Huang CC . Gonadal dysfunction in mitochondrial encephalomyopathies. Eur Neurol. (1995) 35:281–6. doi: 10.1159/000117150, PMID: 8542917

[154] Carod-Artal FJ Herrero MD Lara MC López-Gallardo E Ruiz-Pesini E Martí R . Cognitive dysfunction and hypogonadotrophic hypogonadism in a Brazilian patient with mitochondrial neurogastrointestinal encephalomyopathy and a novel *ECGF1* mutation. Euro J Neurology. (2007) 14:581–5. doi: 10.1111/j.1468-1331.2007.01720.x, PMID: 17437622

[155] Ohkoshi N Ishii A Shiraiwa N Shoji S Yoshizawa K . Dysfunction of the hypothalamic-pituitary system in mitochondrial encephalomyopathies. J Med. (1998) 29:13–29., PMID: 9704289

